# Multi-Target Data Association Algorithm in Underwater BOT System with Spatial Bias and Signal Delay

**DOI:** 10.3390/biomimetics11070489

**Published:** 2026-07-11

**Authors:** Naifu Luo, Hongjian Wang, Zhenwei Lu, Xinyang Li, Jingfei Ren

**Affiliations:** College of Intelligent Systems Science and Engineering, Harbin Engineering University, Harbin 150001, China

**Keywords:** bearing-only tracking, bio-inspiration, data association, sensor bias compensation, expectation maximization

## Abstract

The advancing perception capabilities of an individual unmanned underwater vehicle (UUV) pose new challenges for multi-target perceptual consistency in underwater bearing-only tracking (BOT) systems. Accurate target state estimation necessitates two key prerequisites: sensor bias compensation and precise data association. The biases encompass both sensor spatial bias and signal propagation delay between the target and sensor. This paper introduces a measurement model that explicitly accounts for these factors. To address the lack of prior target information, an initial target state estimation algorithm is developed based on maximum likelihood estimation (MLE), with a refined bio-inspired variant incorporating particle swarm optimization (PSO). To this end, a cost function is formulated to transform the BOT data association problem into an assignment problem. Thereafter, an iterative multi-target data association (MDA) algorithm, integrated with the expectation-maximization (EM) method, is designed to jointly mitigate the effects of signal delay and spatial bias. Monte Carlo simulation scenarios validate the overall effectiveness of the proposed MDA framework. Specifically, the EM-based spatial bias estimation method demonstrates accurate bias estimation capability.

## 1. Introduction

With the rapid development of underwater detection technology, the autonomous capabilities of an unmanned underwater vehicle (UUV) are becoming increasingly critical [1,2]. Using passive sonar as the primary sensing method and bearing-only target tracking as the core technical feature, the UUV can perform sensing and maneuvering operations to track uncooperative targets, which represents a key aspect of the UUV’s autonomous capabilities.

Bearing information of the target is the only measurement that can be acquired by the observation platform in the bearing-only tracking (BOT) scenario. Due to the lack of direct measurements of key parameters such as range and velocity, the target state estimation accuracy is low if the observation platform does not maneuver [3,4]. When the observation platform maneuvers to track multiple targets, the measurement data association becomes challenging due to the fact that the measurement information for different targets is not aligned within a global coordinate system. In addition, spatial bias from the sensor, signal propagation delay between the target and sensor, and the target’s initial state uncertainty introduce significant challenges for data association in an underwater BOT system.

Registration errors refer to inaccuracies arising from various uncertainties in sensors, including asynchronous clocks, imprecise sensor locations, measurement biases and ambiguities in sensor configuration parameters [5]. There are two kinds of biases that impact multi-target tracking precision. One arises from signal propagation delay, which is frequently ignored in BOT problems [6]. In systems like radar or satellite navigation, the emitted signals are electromagnetic waves whose speed is nearly equal to the speed of light propagation in air [7]. Hence, the impact of the propagation delay on tracking performance can be considered negligible. However, in the case of passive acoustic sensors, if the signal propagation delay is neglected, the target is assumed to be at the same position from which the signal was originally emitted. It can lead to substantial discrepancies between the estimated and actual positions of the target [8,9]. Because the target is still moving in reality during the signal propagation from the target to the sensor, the impact of the signal propagation delay on target tracking accuracy has been theoretically investigated in scenarios where the signal travels several orders of magnitude faster than the target [10]. The results indicate that neglecting this delay can result in a significant decline in tracking performance. The other kind of bias is the spatial bias that is caused by a systematic error rather than random noise contained in sensor measurements [11]. Sensor measurement bias can subsequently lead to tracking errors.

Considering the aforementioned registration problems, an appropriate data association algorithm is crucial as a prerequisite for the Kalman filter (KF) and its derivatives to accurately estimate the target state. In [12], a distributed maximum likelihood estimation (MLE) method is adopted for target tracking, which indirectly eliminates sensor position biases by leveraging measurement differences from multiple sensors observing the same target. However, the coordinate transformation increases computational complexity. Due to the lack of consideration for non-linear measurement consistency in the sensor network, the method cannot be applied to multi-target tracking scenarios. In [13], data association is resolved implicitly through geometric consistency in multi-target densities. A cross-sensor target association hypothesis is obtained by maximizing the instantaneous reward factor (IRF) directly. Multi-hypothesis filtering then robustly fuses instantaneous estimates, eliminating ambiguous associations to converge on the drift and orientation consensus. In [14], a geometry-based data association method is introduced, which optimizes camera–radar correspondences through roll–pitch parameter pairs and solves a k-cardinality assignment problem to ensure multi-target consistency. However, its performance remains sensitive to uncalibrated heading offsets. A decentralized evolutionary method (DeEvo) based on bi-level optimization is proposed in [15], where differential evolution and the Kuhn–Munkres algorithm are employed to optimize target locations and data association, respectively, and a momentum-based consensus strategy is designed for co-evolution among sensors. However, DeEvo relies on bearing-line intersections from multiple sensors at different spatial positions to locate targets, while a single maneuvering platform provides only one observation perspective, and bearing-only measurements alone cannot independently resolve target range information, rendering the localization problem inherently underdetermined and leaving the upper-level differential evolution algorithm without effective constraints or observability support in the continuous search space. In [16], a joint probabilistic data association filter (JPDAF) tracker for a bearing-only mobile sensor is proposed, which could handle measurement merging via graph-based modeling. While validated on a real unmanned ground vehicle (UGV) with vision-based bearing measurements, the method relies on the accuracy of initialization during the association process. In [17], an adaptive innovation sequence-based joint probabilistic data association (AIS-JPDA) algorithm is further proposed, in which the confirmation matrix is reconstructed to simplify association probability computation and enhance real-time tracking performance under dense clutter. However, the AIS-JPDA algorithm assumes that radar provides both range and azimuth observations, making it unable to handle the incomplete measurement problem arising from bearing-only observations. In [18], a nonparametric data association and tracking algorithm (NPDAT) is proposed for multistatic radars, which leverages the geometric diversity of distributed transmitter–receiver pairs along with TOA and bistatic Doppler measurements to achieve hierarchical deghosting and tracking with lower computational complexity than assignment-based methods. However, the core localization mechanism of NPDAT fundamentally depends on the multi-site geometric structure provided by spatially distributed transmitter–receiver pairs to resolve target range information. A novel method using probability hypothesis density (PHD) filters and generalized covariance intersection (GCI) divergence is proposed to achieve joint sensor registration and multi-object tracking [19]. A pairwise registration strategy is introduced to decompose the high-dimensional optimization problem into more manageable low-dimensional sub-problems. To address the non-convexity arising from disassociated components, a two-step optimization framework is developed to enhance both efficiency and robustness. In [20], an MLE-based track-to-track association method for T/R-R composite compact HFSWR that resolves measurement uncertainties in multi-target scenarios was introduced. Although the measurement bias is considered, no explicit sensor bias compensation model is incorporated. This method cannot be extended to associate multi-target tracks from a single sensor. In [21], CLIPPER introduces the Densest Edge-Weighted Clique (DEWC) formulation, which utilizes continuous pairwise consistency scores along with clique constraints to enhance association accuracy and ensure global consistency in the tracking framework. The limitation lies in its reliance on a problem-specific geometric invariant, which restricts its ability to generalize to non-linear transformations. Given the mutual influence among data association, registration and fusion processes, several joint association and fusion methods at the measurement level have been proposed to improve overall tracking accuracy and consistency. By minimizing the dual Mahalanobis distance, a joint optimization method is constructed for fused state estimation and dual-sensor bias compensation [22]. The proposed method realizes the collaborative optimization of association and registration, which effectively addresses the coupling problem between sensor biases and data association under a cluttered environment. Considering the spatiotemporal bias of asynchronous sensors, a joint association and fusion algorithm with bias compensation is proposed by integrating feedback mechanisms based on the unscented transformation (UT) with covariance intersection (CI) [23].

The aforementioned method relies on a key assumption that range information from sensor measurements is available, or that a relatively accurate initial target state can be obtained. However, the target’s initial position or other prior information is typically unavailable in a BOT system. Several researchers have focused their efforts on tackling the challenge posed by the absence of range measurements in passive tracking scenarios [24]. Some methods, as mentioned in [25], estimate the target’s trajectory by incorporating frequency information. However, the frequency emitted by the target is subject to fluctuations, and environmental noise can further interfere, which would reduce the reliability of such approaches in engineering applications. MLE is one of the most widely used batch processing techniques, which could be employed to estimate the initial state of the target [26,27]. In [28], an innovative approach is introduced by incorporating partial prior range information into passive target motion analysis and treating it as a pseudo-measurement within an MLE framework. However, the robustness of this method diminishes when prior range assumptions are significantly biased. In underwater BOT scenarios, the initial target state is commonly estimated by batch processing methods. This initial estimate is then used to initialize recursive estimation methods. Such a combined strategy enhances the tracking accuracy and improves the robustness of the recursive approach.

This paper focuses on achieving high precision multi-target motion state estimation using bearing-only measurements from a UUV, while compensating for sensor spatial bias and signal propagation delay. The core challenge addressed is the data association problem in multi-target tracking. The main contributions of this paper are summarized as follows:

1. To address the inherent measurement deviations of passive sonar in underwater environments, a measurement model that incorporates the effects of spatial bias and signal propagation delay is constructed.
2. To overcome the lack of prior information regarding the initial state of targets in bearing-only tracking, a maximum likelihood estimation algorithm based on signal delay compensation is proposed for initial state estimation. Building upon this, an initial target state estimation algorithm based on particle swarm optimization (PSO) is designed to further enhance the accuracy of initial state estimation, which is subsequently applied to the initialization phase of the multi-target tracking process.
3. A cost function is formulated to transform the data association problem in bearing-only tracking with sensor bias into an assignment problem for solution. Furthermore, to address scenarios with unknown spatial bias, an iterative target state estimation algorithm integrating the expectation-maximization (EM) method is proposed. This algorithm mitigates the influence of sensor spatial bias to accurately estimate the target states.
4. The effectiveness of the proposed multi-target data association algorithm is validated through Monte Carlo simulation scenarios. Additionally, the EM-based spatial bias estimation method strikes an effective balance between estimation accuracy and computational efficiency, ensuring real-time algorithm operation.

The remainder of this paper is organized as follows. It begins with Section 2, which illustrates the problem of spatial bias and signal delay in BOT and presents the corresponding multi-target state and measurement models. Section 3 follows by detailing the proposed initial target state estimation method. Subsequently, Section 4 describes the data association and state estimation algorithm. The results and analysis of the simulation scenarios are presented in Section 5. This paper concludes with Section 6, which summarizes this work and outlines future research directions.

## 2. Model Establishment with Spatial Bias and Signal Delay

### 2.1. State Model

For the underwater multi-target BOT system, the *j*th target’s state at time step *k* is given by

(1) $$X_{j}\left(k\right)={\left[x_{j}\left(k\right),y_{j}\left(k\right),{\dot{x}}_{j}\left(k\right),{\dot{y}}_{j}\left(k\right)\right]}^{T},\;j=1,\cdots ,J,$$

where $\left[x_{j}\left(k\right),y_{j}\left(k\right)\right]$ and $\left[{\dot{x}}_{j}\left(k\right),{\dot{y}}_{j}\left(k\right)\right]$ represent the two-dimensional position and velocity information of the *j*th target in the north-east-down (NED) coordinate. The coordinate transformation from the target’s frame to the NED coordinate follows the description in [29].

The state model of the *j*th target is described as

(2) $$X_{j}\left(k\right)=F\left(k\right)X_{j}\left(k-1\right)+U\left(k\right),$$

where $U\left(k\right)$ represents the process error, which is assumed to be zero-mean Gaussian noise with the variance matrix $Q\left(k\right)$. It can be written as

(3) $$Q\left(k\right)=qI_{4\times 4}\Gamma ,\;I_{4\times 4}≜\left(\begin{array}{cccc} 1 & 0 & 0 & 0\\ 0 & 1 & 0 & 0\\ 0 & 0 & 1 & 0\\ 0 & 0 & 0 & 1 \end{array}\right),$$

where $\Gamma ={\left[\begin{array}{c} \frac{\Delta {T_{U}}^{2}}{2},\frac{\Delta {T_{U}}^{2}}{2},\Delta T_{U},\Delta T_{U} \end{array}\right]}^{T}$ is the noise driving matrix. *q* represents the power spectral density of the process noise. $\Delta T_{U}$ is the sampling interval of the UUV. The target’s motion is modeled with a constant velocity (CV) model. The state transition matrix $F\left(k\right)$ can be expressed as

(4) $$F\left(k\right)=\left(\begin{array}{cc} I_{2\times 2} & \Delta T_{U}I_{2\times 2}\\ 0 & I_{2\times 2} \end{array}\right),\;I_{2\times 2}≜\left(\begin{array}{cc} 1 & 0\\ 0 & 1 \end{array}\right).$$

### 2.2. Measurement Model

The UUV’s state can be written as $X_{U}\left(k\right)={\left[x\left(k\right),y\left(k\right),\dot{x}\left(k\right),\dot{y}\left(k\right)\right]}^{T}$, where $\left[x\left(k\right),y\left(k\right)\right]$ and $\left[\dot{x}\left(k\right),\dot{y}\left(k\right)\right]$ represent the UUV’s position and velocity in the NED coordinate. This paper assumes that the *i*th measurement from the UUV at time step *k* is denoted as $z_{U}^{i}\left(k\right)$, and this measurement corresponds to the *j*th target’s state. If the measurement originates from the target $X_{j}\left(k\right)$, then the measurement model can be described by the following equation:

(5) $$\begin{array}{cc} z_{U}^{i}\left(k\right) & =h\left(X_{U}\left(k\right),X_{j}\left(k\right)\right)+\Delta \theta_{U}^{i}\left(k\right)+V\left(k\right)\\ \; & =\arctan\left(\frac{x_{j}\left(k\right)-{\dot{x}}_{j}\left(k\right)\Delta \tau_{U}^{i}\left(k\right)-x\left(k\right)}{y_{j}\left(k\right)-{\dot{y}}_{j}\left(k\right)\Delta \tau_{U}^{i}\left(k\right)-y\left(k\right)}\right)+\Delta \theta_{U}^{i}\left(k\right)+V\left(k\right) \end{array}.$$

$\Delta \tau_{U}^{i}\left(k\right)$ and $\Delta \theta_{U}^{i}\left(k\right)$∼$N\left(\Delta {\bar{\theta }}_{U},R_{U}\right)$ represent the signal delay and spatial bias of the UUV’s *i*th measurement at time step *k*, respectively.

If the measurement originates from clutter, then the measurement model can be written as

(6) $$z_{U}^{i}\left(k\right)={\tilde{z}}_{U}^{i}\left(k\right).$$

The probability density function of ${\tilde{z}}_{U}^{i}\left(k\right)$ is given by

(7) $$p\left({\tilde{z}}_{U}^{i}\left(k\right)\right)=1/\Psi_{U},\;$$

where $\Psi_{U}$ represents the volume of the passive sonar’s field of view.

As mentioned in Section 1, the measurement time is not exactly the same as the time corresponding to the target’s state. The signal delay between the target and the UUV is taken into account in Equation (5).

## 3. Initial Target State Estimation

### 3.1. Rough Initial Target State Estimation Based on MLE

Accounting for the impact of the signal propagation delay, this paper employs MLE to estimate the rough initial target state. Assuming that the *i*th measurement originating from the *j*th target follows a Gaussian distribution, the likelihood function is given by

(8) $$\begin{array}{cc} p & \left(z_{U}^{i}|{\hat{X}}_{j}\right)={\left[{\left(2\pi \right)}^{K_{i}}\det R_{U}\right]}^{-\frac{1}{2}}\exp\left[-\frac{1}{2}{\left(z_{U}^{i}-h\left(X_{U},{\hat{X}}_{j}\right)\right)}^{T}R_{U}^{-1}\left(z_{U}^{i}-h\left(X_{U},{\hat{X}}_{j}\right)\right)\right] \end{array},$$

where $z_{U}^{i}$ and $h\left(X_{U},{\hat{X}}_{j}\right)$ represent the stack forms of acquired and predicted measurements, which can be written as

(9) $$\begin{array}{cc} z_{U}^{i}= & {\left[z_{U}^{i}\left(1\right),\cdots ,z_{U}^{i}\left(K_{i}\right)\right]}^{T}\\ h\left(X_{U},{\hat{X}}_{j}\right)= & {\left[h\left(X_{U}\left(1\right),{\hat{X}}_{j}\left(1\right)\right),\cdots ,h\left(X_{U}\left(K_{i}\right),{\hat{X}}_{j}\left(K_{i}\right)\right)\right]}^{T}. \end{array}$$

The variance matrix of the measurement error is given by $R_{U}=R_{U}I_{K_{i}\times K_{i}}$. $K_{i}$ is the total time steps of the measurements corresponding to *j*th target.

To maximize the likelihood function Equation (8), estimation of the initial target state is formulated as

(10) $${\hat{X}}_{j}\left(1\right)=\underset{{\hat{X}}_{j}\left(1\right)}{\arg\max}\;p\left(z_{U}^{i}|{\hat{X}}_{j}\right).$$

Taking the derivative of Equation (10) with respect to ${\hat{X}}_{j}\left(1\right)$, the solution can be written as

(11) $${\left[\frac{\partial h\left(X_{U},{\hat{X}}_{j}\right)}{\partial {\hat{X}}_{j}\left(1\right)}\right]}^{T}R_{U}^{-1}\left(z_{U}^{i}-h\left(X_{U},{\hat{X}}_{j}\right)\right)=0.$$

The partial derivative of the predicted measurement with respect to the *l*th element $\left(l=1,2,3,4\right)$ of the estimated target state ${\hat{X}}_{j}\left(1\right)$ can be expressed as

(12) $$\begin{array}{cc} \; & \frac{\partial h\left(X_{U}\left(k\right),{\hat{X}}_{j}\left(k\right)\right)}{\partial {\hat{X}}_{j}\left(1\right)\left[l\right]}=\frac{1}{D_{U}\left(k\right)}\left[\cos\left(z_{U}^{i}\left(k\right)\right)\frac{\partial {\hat{x}}_{j}\left(k-\Delta \tau_{U}^{i}\left(k\right)\right)}{\partial {\hat{X}}_{j}\left(1\right)\left[l\right]}-\sin\left(z_{U}^{i}\left(k\right)\right)\frac{\partial {\hat{y}}_{j}\left(k-\Delta \tau_{U}^{i}\left(k\right)\right)}{\partial {\hat{X}}_{j}\left(1\right)\left[l\right]}\right] \end{array},$$

where

(13) $$\begin{array}{cc} \; & \begin{array}{cc} D_{U}\left(k\right) & =\left({\left({\hat{x}}_{j}\left(k-\Delta \tau_{U}^{i}\left(k\right)\right)-x\left(k\right)\right)}^{2}\right.{\left.+{\left({\hat{y}}_{j}\left(k-\Delta \tau_{U}^{i}\left(k\right)\right)-y\left(k\right)\right)}^{2}\right)}^{\frac{1}{2}}\\ \; & =c\Delta \tau_{U}^{i}\left(k\right) \end{array}\\ \; & {\hat{x}}_{j}\left(k-\Delta \tau_{U}^{i}\left(k\right)\right)={\hat{x}}_{j}\left(k\right)-{\hat{\dot{x}}}_{j}\left(k\right)\Delta \tau_{U}^{i}\left(k\right)\\ \; & {\hat{y}}_{j}\left(k-\Delta \tau_{U}^{i}\left(k\right)\right)={\hat{y}}_{j}\left(k\right)-{\hat{\dot{y}}}_{j}\left(k\right)\Delta \tau_{U}^{i}\left(k\right) \end{array}.$$

*c* represents the measured speed of underwater sound signal propagation. The detailed expressions for each derivative of Equation (12) are derived as follows:

(14) $$\begin{array}{cc} \frac{\partial {\hat{x}}_{j}\left(k-\Delta \tau_{U}^{i}\left(k\right)\right)}{\partial {\hat{x}}_{j}\left(k\right)} & =1-{\hat{\dot{x}}}_{j}\left(k\right)\frac{\partial \Delta \tau_{U}^{i}\left(k\right)}{\partial {\hat{x}}_{j}\left(k\right)}\\ \frac{\partial {\hat{y}}_{j}\left(k-\Delta \tau_{U}^{i}\left(k\right)\right)}{\partial {\hat{x}}_{j}\left(k\right)} & =-{\hat{\dot{y}}}_{j}\left(k\right)\frac{\partial \Delta \tau_{U}^{i}\left(k\right)}{\partial {\hat{x}}_{j}\left(k\right)}\\ \frac{\partial {\hat{x}}_{j}\left(k-\Delta \tau_{U}^{i}\left(k\right)\right)}{\partial {\hat{\dot{x}}}_{j}\left(k\right)} & =k-\Delta \tau_{U}^{i}\left(k\right)-{\hat{\dot{x}}}_{j}\left(k\right)\frac{\partial \Delta \tau_{U}^{i}\left(k\right)}{\partial {\hat{\dot{x}}}_{j}\left(k\right)}\\ \frac{\partial {\hat{y}}_{j}\left(k-\Delta \tau_{U}^{i}\left(k\right)\right)}{\partial {\hat{\dot{x}}}_{j}\left(k\right)} & =-{\hat{\dot{y}}}_{j}\left(k\right)\frac{\partial \Delta \tau_{U}^{i}\left(k\right)}{\partial {\hat{\dot{x}}}_{j}\left(k\right)}\\ \frac{\partial {\hat{y}}_{j}\left(k-\Delta \tau_{U}^{i}\left(k\right)\right)}{\partial {\hat{y}}_{j}\left(k\right)} & =1-{\hat{\dot{y}}}_{j}\left(k\right)\frac{\partial \Delta \tau_{U}^{i}\left(k\right)}{\partial {\hat{y}}_{j}\left(k\right)}\\ \frac{\partial {\hat{x}}_{j}\left(k-\Delta \tau_{U}^{i}\left(k\right)\right)}{\partial {\hat{y}}_{j}\left(k\right)} & =-{\hat{\dot{x}}}_{j}\left(k\right)\frac{\partial \Delta \tau_{U}^{i}\left(k\right)}{\partial {\hat{y}}_{j}\left(k\right)}\\ \frac{\partial {\hat{x}}_{j}\left(k-\Delta \tau_{U}^{i}\left(k\right)\right)}{\partial {\hat{\dot{y}}}_{j}\left(k\right)} & =-{\hat{\dot{x}}}_{j}\left(k\right)\frac{\partial \Delta \tau_{U}^{i}\left(k\right)}{\partial {\hat{\dot{y}}}_{j}\left(k\right)}\\ \frac{\partial {\hat{y}}_{j}\left(k-\Delta \tau_{U}^{i}\left(k\right)\right)}{\partial {\hat{\dot{y}}}_{j}\left(k\right)} & =k-\Delta \tau_{U}^{i}\left(k\right)-{\hat{\dot{y}}}_{j}\left(k\right)\frac{\partial \Delta \tau_{U}^{i}\left(k\right)}{\partial {\hat{\dot{y}}}_{j}\left(k\right)} \end{array}.$$

Since the partial derivatives of Equation (14) involve signal delay, the computation result of these parts can be expressed as

(15) $$\begin{array}{cc} \frac{\partial \Delta \tau_{U}^{i}\left(k\right)}{\partial {\hat{x}}_{j}\left(1\right)} & =\frac{\sin\left(z_{U}^{i}\left(k\right)\right)}{c+{\hat{\dot{x}}}_{j}\left(k\right)\sin\left(z_{U}^{i}\left(k\right)\right)+{\hat{\dot{y}}}_{j}\left(k\right)\cos\left(z_{U}^{i}\left(k\right)\right)}\\ \frac{\partial \Delta \tau_{U}^{i}\left(k\right)}{\partial {\hat{y}}_{j}\left(1\right)} & =\frac{\cos\left(z_{U}^{i}\left(k\right)\right)}{c+{\hat{\dot{x}}}_{j}\left(k\right)\sin\left(z_{U}^{i}\left(k\right)\right)+{\hat{\dot{y}}}_{j}\left(k\right)\cos\left(z_{U}^{i}\left(k\right)\right)}\\ \frac{\partial \Delta \tau_{U}^{i}\left(k\right)}{\partial {\hat{\dot{x}}}_{j}\left(1\right)} & =\left[k-\Delta \tau_{U}^{i}\left(k\right)\right]\frac{\partial \Delta \tau_{U}^{i}\left(k\right)}{\partial {\hat{x}}_{j}\left(1\right)}\\ \frac{\partial \Delta \tau_{U}^{i}\left(k\right)}{\partial {\hat{\dot{y}}}_{j}\left(1\right)} & =\left[k-\Delta \tau_{U}^{i}\left(k\right)\right]\frac{\partial \Delta \tau_{U}^{i}\left(k\right)}{\partial {\hat{y}}_{j}\left(1\right)} \end{array}.$$

Then, substituting Equation (15) into Equation (12), the partial derivatives are computed with the resulting expression, given by

(16) $$\begin{array}{cc} \frac{\partial h\left(X_{U}\left(k\right),{\hat{X}}_{j}\left(k\right)\right)}{\partial {\hat{x}}_{j}\left(1\right)} & =\frac{\left\{\cos\left(z_{U}^{i}\left(k\right)\right)+\Lambda \sin\left(z_{U}^{i}\left(k\right)\right)\right\}}{D_{U}\left(k\right)}\\ \frac{\partial h\left(X_{U}\left(k\right),{\hat{X}}_{j}\left(k\right)\right)}{\partial {\hat{y}}_{j}\left(1\right)} & =\frac{\left\{-\sin\left(z_{U}^{i}\left(k\right)\right)+\Lambda \cos\left(z_{U}^{i}\left(k\right)\right)\right\}}{D_{U}\left(k\right)}\\ \frac{\partial h\left(X_{U}\left(k\right),{\hat{X}}_{j}\left(k\right)\right)}{\partial {\hat{\dot{x}}}_{j}\left(1\right)} & =\kappa \frac{\partial h\left(X_{U}\left(k\right),{\hat{X}}_{j}\left(k\right)\right)}{\partial {\hat{x}}_{j}\left(k\right)}\\ \frac{\partial h\left(X_{U}\left(k\right),{\hat{X}}_{j}\left(k\right)\right)}{\partial {\hat{\dot{y}}}_{j}\left(1\right)} & =\kappa \frac{\partial h\left(X_{U}\left(k\right),{\hat{X}}_{j}\left(k\right)\right)}{\partial {\hat{y}}_{j}\left(k\right)} \end{array},$$

where $\Lambda =\frac{{\hat{\dot{y}}}_{j}\left(k\right)\sin\left(z_{U}^{i}\left(k\right)\right)-{\hat{\dot{x}}}_{j}\left(k\right)\cos\left(z_{U}^{i}\left(k\right)\right)}{c+{\hat{\dot{x}}}_{j}\left(k\right)\sin\left(z_{U}^{i}\left(k\right)\right)+{\hat{\dot{y}}}_{j}\left(k\right)\cos\left(z_{U}^{i}\left(k\right)\right)}$ and $\kappa =\left[k\Delta T_{U}-\sum_{n=1}^{k}\Delta \tau_{U}^{i}\left(n\right)\right]$.

Recall the component of $\left[\frac{\partial h\left(X_{U},{\hat{X}}_{j}\right)}{\partial {\hat{X}}_{j}\left(1\right)}\right]$ in Equation (11); it can be denoted as

(17) $$A={\left[\begin{array}{ccc} \frac{\partial h\left(X_{U}\left(1\right),{\hat{X}}_{j}\left(1\right)\right)}{\partial {\hat{X}}_{j}\left(1\right)} & \cdots & \frac{\partial h\left(X_{U}\left(K_{i}\right),{\hat{X}}_{j}\left(K_{i}\right)\right)}{\partial {\hat{X}}_{j}\left(1\right)} \end{array}\right]}^{T}.$$

A Gauss–Newton iteration scheme is adopted to compute the solution of MLE Equation (10). The iterative equation can be written as

(18) $$\begin{array}{cc} {\left({\hat{X}}_{j}\left(1\right)\right)}^{iter+1}= & {\left({\hat{X}}_{j}\left(1\right)\right)}^{iter}+\delta_{iter}{\left[A^{T}{R_{U}}^{-1}A\right]}^{-1}A^{T}{R_{U}}^{-1}\left(z_{U}^{i}-h\left(X_{U},{\hat{X}}_{j}\right)\right) \end{array},$$

where *δ_iter_* denotes the step size at the iterth iteration.

### 3.2. Precise Initial Target State Estimation Based on PSO

The Jacobian matrix A of Equation (17) describes the influence of estimated initial target state changes on the range and velocity residuals. The direction of the Gauss–Newton iteration is perpendicular to the line of sight, the bearing direction from the UUV to the target, which is the direction in which bearing residuals are most sensitive to changes in the target state. Furthermore, the algorithm is highly sensitive to the choice of the initial target state. Due to the absence of prior information, if the estimation of the initial target state deviates significantly from the true value, the algorithm may converge to a local minimum or even diverge entirely. Therefore, this paper adopts the PSO algorithm with MLE to obtain a precise estimation of the initial target state.

The optimization function is defined as

(19) $$\min f=\sqrt{\sum_{k=1}^{K_{i}}{\left(z_{U}^{i}\left(k\right)-h\left(X_{U}\left(k\right),{\hat{X}}_{j}\left(k\right)\right)\right)}^{2}}.$$

To exploit the target’s bearing information and reduce the optimization search space, the initial position of the target is optimized with the proposed signal delay compensated MLE through gradient-based search, while the target’s initial velocity and course are heuristically optimized using the PSO algorithm proposed, as follows.

The position of the particle is defined as

(20) $$p_{l}^{iter}={\left[\begin{array}{cc} V_{l}^{iter} & \psi_{l}^{iter} \end{array}\right]}^{T},\;1\leq l\leq L,$$

where *L* denotes the population size of the particles in the swarm. $V_{l}^{iter}$ and $\psi_{l}^{iter}$ denote the target’s initial velocity and course, respectively.

The velocity of particle $p_{l}^{iter}$ can be written as

(21) $$v_{l}^{iter}={\left[\begin{array}{cc} \upsilon_{V}^{iter} & \upsilon_{\psi }^{iter} \end{array}\right]}^{T},\;1\leq l\leq L.$$

To update the particle velocity, the operator is designed as

(22) $$\begin{array}{cc} v_{l}^{iter+1}= & \alpha^{iter}⊙v_{l}^{iter}+\beta^{iter}⊙r_{1}^{iter}\left(p_{l}-p_{l}^{iter}\right)+\gamma^{iter}⊙r_{2}^{iter}\left(p_{g}-p_{l}^{iter}\right) \end{array},$$

where

(23) $$\begin{array}{cc} \alpha^{iter} & =\frac{N_{iter}-iter}{N_{iter}+0.4}\left(\alpha^{iter-1}-{\left[\begin{array}{cc} 0.4 & 0.4 \end{array}\right]}^{T}\right)\\ \beta^{iter} & =\left(\beta^{\min}-\beta^{\max}\right)\frac{iter}{N_{iter}}+\beta^{\max}\\ \gamma^{iter} & =\left(\gamma^{\max}-\gamma^{\min}\right)\frac{iter}{N_{iter}}+\gamma^{\min} \end{array}.$$

⊙ denotes the Hadamard product. $p_{l}$ denotes the individual best particle. $p_{g}$ denotes the global best particle. $\alpha^{iter}={\left[\begin{array}{cc} \alpha_{V}^{iter} & \alpha_{\psi }^{iter} \end{array}\right]}^{T}$ represents the inertia weight of the algorithm. $\beta^{iter}={\left[\begin{array}{cc} \beta_{V}^{iter} & \beta_{\psi }^{iter} \end{array}\right]}^{T}$ and $\gamma^{iter}={\left[\begin{array}{cc} \gamma_{V}^{iter} & \gamma_{\psi }^{iter} \end{array}\right]}^{T}$ represent the acceleration coefficients of the algorithm. $\beta^{\max}$ and $\gamma^{\max}$ denote the maximum values of the acceleration coefficients. $\beta^{\min}$ and $\gamma^{\min}$ denote the minimum values of the acceleration coefficients. $r_{1}^{iter}$ and $r_{2}^{iter}$ are random numbers uniformly drawn from the interval [0,1], introduced to incorporate uncertainty into the algorithm. $N_{iter}$ denotes the maximum number of iterations of the algorithm.

The constraints of the particles are summarized as follows:

(24) $$\begin{array}{cc} 0 & \leq V_{l}^{iter}\leq v_{j\max}\\ 0 & \leq \psi_{l}^{iter}<360 \end{array},$$

where $v_{j\max}$ represents the maximum velocity of the *j*th target.

Considering that the search space for the target’s course is larger than that for velocity, the coupling factor is introduced to enhance the exploration capability in the course dimension while moderately suppressing exploration in the velocity dimension, thereby achieving improved algorithmic performance.

(25) $$v_{l}^{iter+1}=v_{l}^{iter+1}+\mu ⊙\left(p_{l}^{iter}-{\bar{p}}_{l}^{iter}\right)$$

$\mu ={\left[\begin{array}{cc} \mu_{V} & \mu_{\psi } \end{array}\right]}^{T}$ denotes the coupling factor. ${\bar{p}}_{l}^{iter}$ represents the mean value of the current particle swarm.

To sum up, the pseudo-code of the proposed PSO algorithm for the precise initial target state estimation described above is presented in Algorithm 1.
**Algorithm 1:** PSO algorithm for precise initial target state estimation
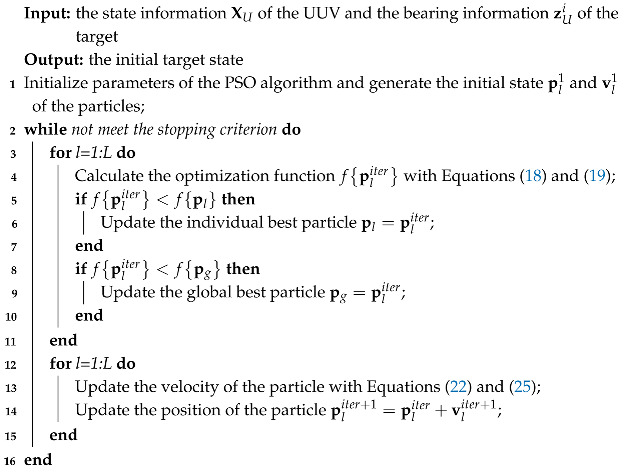


## 4. Data Association and State Estimation

### 4.1. Measurement Association

Refer to [30,31]; the BOT system becomes observable when the motion order of the observation platform is higher than that of the target. When the observation platform maneuvers to track multiple targets, the challenge lies in correctly associating measurements with different targets, because the bearing measurements are defined in the observation space, whereas the target states are described in the NED coordinate space.

The measurement–track pair is defined as ${\{}_{U}^{i}\left(k\right),j\}$, which consists of the *i*th measurement of the UUV and the *j*th target at time step *k*. The cost function of a measurement–track pair is designed as the negative logarithm of the likelihood ratio.

(26) c,Uijk=−lnpzUik|X^jkpzUik|j=0=εi−1ln1−PDi+εilnPDiΨU−1NzUik;hXUk,X^jk,RU

The measurement obtained from the spurious source is denoted as $p\left(z_{U}^{i}\left(k\right)|j=0\right)$. The successful detection probability of the UUV is denoted as $P_{D}^{i}$. $\varepsilon_{i}$ is the binary indicator. When the measurement from target $z_{U}^{i}$ is detected, $\varepsilon_{i}$ is equal to one. Otherwise, $\varepsilon_{i}=0$.

The Mahalanobis distance is defined as

(27) $$D{\{}_{U}^{i}\left(k\right),j\}={\tilde{z}}_{U}^{i}\left(k\right){R_{U}}^{-1}{\tilde{z}}_{U}^{i}\left(k\right),$$

where ${\tilde{z}}_{U}^{i}\left(k\right)=z_{U}^{i}\left(k\right)-h\left(X_{U}\left(k\right),{\hat{X}}_{j}\left(k\right)\right)$. The validation gate is denoted as Υ. If $D{\{}_{U}^{i}\left(k\right),j\}\leq \Upsilon$, the cost function is calculated by Equation (26). Otherwise, c,Uijk=inf, which means the measurement–track pair is not taken into consideration.

The minimization of the global association cost can be expressed as follows:

(28) minωi,j∑j=1Ntk−1∑i=1Nikc,Uijkωi,jk,

subject to

(29) $$\begin{array}{cc} \; & \sum_{i=1}^{N_{i}\left(k\right)}\omega_{i,j}\left(k\right)\leq 1,\;j=1,\cdots ,N_{t}\left(k-1\right)\\ \; & \sum_{j=1}^{N_{t}\left(k-1\right)}\omega_{i,j}\left(k\right)\leq 1,\;i=1,\cdots ,N_{i}\left(k\right) \end{array},$$

where $\omega_{i,j}\left(k\right)$ is the 0−1 binary indicator. $N_{t}\left(k-1\right)$ is denoted as the number of targets at time step k−1. $N_{i}\left(k\right)$ represents the number of measurements received by the UUV at time step *k*.

### 4.2. Target State Estimation with Spatial Bias Compensation

The state estimation for each target track is recursively updated using the measurements from the UUV. First, data association is performed as formulated in Equation (28), and for each measurement–track pair ${\{}_{U}^{i}\left(k\right),j\}$, the state estimate is subsequently refined via an extended Kalman filter (EKF) with the consideration of the signal delay.

(30) $$\begin{array}{cc} {\hat{X}}_{j}\left(k,k-1\right)= & F\left(k\right){\hat{X}}_{j}\left(k-1,k-1\right)\\ P_{j}\left(k,k-1\right)= & F\left(k\right)P_{j}\left(k-1,k-1\right)F^{T}\left(k\right)+Q\left(k\right)\\ K_{j}\left(k\right)= & P_{j}\left(k,k-1\right){H_{j}}^{T}\left(k\right){\left[H_{j}\left(k\right)P_{j}\left(k,k-1\right){H_{j}}^{T}\left(k\right)+R_{U}\left(k\right)\right]}^{-1}\\ {\hat{X}}_{j}\left(k,k\right)= & {\hat{X}}_{j}\left(k,k-1\right)+K_{j}\left(k\right)\left[z_{U}^{i}\left(k\right)-h\left(X_{U}\left(k\right),{\hat{X}}_{j}\left(k,k-1\right)\right)\right]\\ P_{j}\left(k,k\right)= & \left[I_{4\times 4}-K_{j}\left(k\right)H_{j}\left(k\right)\right]P_{j}\left(k,k-1\right) \end{array}$$

The Jacobian of the measurement function is denoted as $Hj\left(k\right)$. Refer to Equation (16); it is given by

(31) $$H_{j}\left(k\right)={\left[\begin{array}{c} \frac{\partial h\left(X_{U}\left(k\right),{\hat{X}}_{j}\left(k,k-1\right)\right)}{\partial {\hat{x}}_{j}\left(k,k-1\right)}\\ \frac{\partial h\left(X_{U}\left(k\right),{\hat{X}}_{j}\left(k,k-1\right)\right)}{\partial {\hat{y}}_{j}\left(k,k-1\right)}\\ \frac{\partial h\left(X_{U}\left(k\right),{\hat{X}}_{j}\left(k,k-1\right)\right)}{\partial {\hat{\dot{x}}}_{j}\left(k,k-1\right)}\\ \frac{\partial h\left(X_{U}\left(k\right),{\hat{X}}_{j}\left(k,k-1\right)\right)}{\partial {\hat{\dot{y}}}_{j}\left(k,k-1\right)} \end{array}\right]}^{T}.$$

In an underwater BOT system, the complete data sequence through total time steps $K_{i}$ comprises two primary components: the complete set of measurements $z_{U}$ and the entire set of target states $X_{1,\cdots ,J}$. Hence, the function of complete data log-likelihood can be written as

(32) $$\begin{array}{c} \begin{array}{cc} L\left(z_{U},X_{1,\cdots ,J}|\Delta \theta_{U}\right) & =\ln p\left(z_{U},X_{1,\cdots ,J}|\Delta \theta_{U}\right)\\ & =\sum_{k=1}^{K_{i}}\ln p\left(z_{U}^{i}\left(k\right)|X_{1,\cdots ,J}\left(k\right),\Delta \theta_{U}\right) \end{array}, \end{array}$$

where

(33) $$\begin{array}{cc} \; & \ln p\left(z_{U}^{i}\left(k\right)|X_{1,\cdots ,J}\left(k\right),\Delta \theta_{U}\right)=\sum_{j=1}^{N_{t}\left(k\right)}\sum_{i=1}^{N_{i}\left(k\right)}\omega_{i,j}\left(k\right)\ln p\left(z_{U}^{i}\left(k\right)|X_{j}\left(k\right),\Delta \theta_{U}^{i}\left(k\right)\right). \end{array}$$

The maximum likelihood estimation of the parameter $\Delta \theta_{U}^{i}$ is obtained via the expectation-maximization (EM) algorithm. This approach is adopted due to the intractability of directly maximizing Equation (32), which involves the unknown variables $X_{1,\cdots ,J}$ and the latent parameter $\Delta \theta_{U}^{i}$. The EM algorithm operates an iterative process comprising the two following steps:

(34) $$\begin{array}{cc} E\text{-}\mathrm{Step}:\; & Q\left(\Delta \theta_{U},\Delta {\theta_{U}}^{iter}\right)=E\left[L\left(z_{U},X_{1,\cdots ,J}|\Delta \theta_{U}\right)|X_{1,\cdots ,J},\Delta {\theta_{U}}^{iter}\right]\\ M\text{-}\mathrm{Step}:\; & \Delta {\theta_{U}}^{iter+1}=\underset{\Delta \theta_{U}}{\arg\max}\;Q\left(\Delta \theta_{U},\Delta {\theta_{U}}^{iter}\right) \end{array}.$$

In the expectation (E) step, the conditional expectation of the function in Equation (33) is evaluated:

(35) $$\begin{array}{cc} \; & E\left[\ln p\left(z_{U}^{i}\left(k\right)|X_{1,\cdots ,J}\left(k\right),\Delta \theta_{U}\right)\right]=\sum_{j=1}^{N_{t}\left(k\right)}\sum_{i=1}^{N_{i}\left(k\right)}\omega_{i,j}\left(k\right)E\left[\ln p\left(z_{U}^{i}\left(k\right)|X_{j}\left(k\right),\Delta \theta_{U}^{i}\right)\right] \end{array},$$

where

(36) $$\begin{array}{cc} \; & E\left[\ln p\left(z_{U}^{i}\left(k\right)|X_{j}\left(k\right),\Delta \theta_{U}^{i}\right)\right]\\ \; & \begin{array}{cc} = & \ln\left(\frac{P_{D}^{i}}{2\pi {R_{U}}^{1/2}}\right)-\frac{1}{2}E\left[\Delta z{\{}_{U}^{i}\left(k\right),j\}{R_{U}}^{-1}\Delta z{\{}_{U}^{i}\left(k\right),j\}\right] \end{array}\\ \; & \begin{array}{cc} = & \ln\left(\frac{P_{D}^{i}}{2\pi {R_{U}}^{1/2}}\right)-\frac{1}{2}\mathrm{Tr}\left\{{R_{U}}^{-1}\left[\left[Hj^{T}\left(k\right)P_{j}\left(k,k\right)Hj\left(k\right)\right]+\Delta z{{\{}_{U}^{i}\left(k\right),j\}}^{2}\right]\right\} \end{array}\\ \; & \Delta z{\{}_{U}^{i}\left(k\right),j\}=z_{U}^{i}\left(k\right)-h\left(X_{U}\left(k\right),{\hat{X}}_{j}\left(k\right)\right)-\Delta \theta_{U}^{iter}. \end{array}$$

In the maximization (M) step, the parameter $\Delta \theta_{U}$ is estimated by maximizing the expectation function from the E-Step (Equation (34)). Given that $\Delta \theta_{U}$ influences the function solely through the term $\Delta z{\{}_{U}^{i}\left(k\right),j\}$ in Equation (35), it implies that upon substituting Equation (35) into Equation (34), the partial derivative of the objective function with respect to $\Delta \theta_{U}$ is expressed as

(37) ∂ELzU,X1,⋯,J|ΔθU|X1,⋯,J,ΔθUiter∂ΔθU=−∑Kik=ki∑Ntkj=1∑Niki=1ωi,jkRU−1Δz{kUi,j}=0,

where $k_{i}\in \left[1,K_{i}\right)$. Then, $\Delta {\theta_{U}}^{iter+1}$ could be derived as

(38) $$\begin{array}{cc} \; & \Delta {\theta_{U}}^{iter+1}=\frac{\underset{k=k_{i}}{\sum^{K_{i}}}\;\underset{j=1}{\sum^{N_{t}\left(k\right)}}\;\underset{i=1}{\sum^{N_{i}\left(k\right)}}\;\omega_{i,j}\left(k\right){R_{U}}^{-1}\left(z_{U}^{i}\left(k\right)-h\left(X_{U}\left(k\right),{\hat{X}}_{j}\left(k\right)\right)\right)}{\underset{k=k_{i}}{\sum^{K_{i}}}\;\underset{j=1}{\sum^{N_{t}\left(k\right)}}\;\underset{i=1}{\sum^{N_{i}\left(k\right)}}\;\omega_{i,j}\left(k\right){R_{U}}^{-1}}. \end{array}$$

### 4.3. Summary of the Proposed MDA Algorithm

This section summarizes the proposed multi-target data association algorithm, which is subsequently referred to as the MDA algorithm, for estimating target states. The complete pseudo-code of the MDA algorithm is consequently outlined in Algorithm 2. As established in [30,31], a sufficient condition for the observability of the BOT system is that the motion order of the observation platform exceeds that of the target. Measurement data acquired by the UUV prior to its maneuver are incorporated for initial target state estimation, in accordance with the methodology detailed in Steps 2 and 3. The processes of data association and state estimation are described from Steps 6 to 7. A strategic approach to maintaining the real-time performance of the MDA algorithm is to execute the EM-based spatial bias estimation (Steps 8–13) as a single batch process, executed only after a sufficient volume of measurement data has been accumulated.
**Algorithm 2:** MDA algorithm
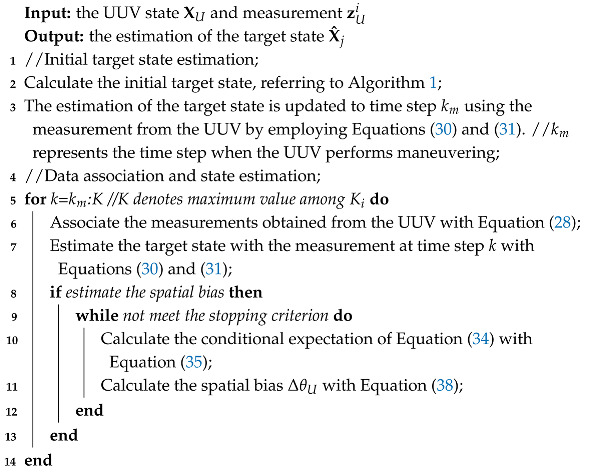


### 4.4. Computational Complexity Analysis

To thoroughly evaluate the practical applicability and real-time deployment potential of the proposed MDA algorithm, this section provides a comprehensive computational complexity analysis. Let *n* denote the dimension of the estimated target state vector ${\hat{X}}_{j}$, $N_{iter}$ denote the maximum iteration number of the PSO algorithm, $N_{EM}$ denote the iteration number of the EM algorithm and $l_{EM}$ denote the sample size of the EM algorithm.

**Phase 1: PSO algorithm for precise initial target state estimation (Algorithm 2, Line 2, invoking Algorithm 1).** At each particle evaluation, the cost function Equation (19) is computed via the Gauss–Newton MLE iteration Equation (18), which entails:

(i) Constructing the $k_{m}\times n$ Jacobian matrix A at cost $O\left(k_{m}\cdot n\right)$;

(ii) Forming the approximate Hessian $A^{T}{R_{U}}^{-1}A$ at cost $O\left(n^{2}\cdot k_{m}\right)$;

(iii) Inverting the resulting n×n matrix at cost $O\left(n^{3}\right)$.

With $N_{GN}$ inner iterations per particle, the single particle cost is $O\left(N_{GN}\cdot \left(n^{2}k_{m}+n^{3}\right)\right)$. Consequently, the overall initial target state estimation stage cost is

(39) $$O\left(N_{t}\cdot N_{iter}\cdot L\cdot N_{GN}\cdot \left(n^{2}k_{m}+n^{3}\right)\right).$$

**Phase 2: Pre-maneuver EKF propagation (Algorithm 2, Line 3).** The EKF propagates the state and covariance of each target from the initial time to the maneuvering time $k_{m}$. A single EKF update consists of several matrix operations, including the state prediction, covariance prediction, Kalman gain computation and covariance update. Although both the covariance prediction and the covariance update involve n×n matrix multiplications, their respective computational costs are summed together. By applying the addition rule for complexity analysis, the highest order term among them, namely, $O\left(n^{3}\right)$, dominates the overall per-step cost. Consequently, performing this propagation for all targets over the $k_{m}$ time steps yields a total complexity of $O\left(N_{t}\cdot k_{m}\cdot n^{3}\right)$.

**Phase 3: Main recursive loop—data association and state estimation (Algorithm 2, Line 5–14).** The main loop runs from the maneuver time $k_{m}$ to the final step *K*, yielding a total of $K-k_{m}+1$ cycles. It is important to highlight that $k_{m}$ is independent of the total time step *K*. Therefore, the difference $K-k_{m}+1$ does not affect the scaling order of the algorithm. Within each cycle, the algorithm sequentially executes data association and EKF updates. Aggregating these per-frame costs and multiplying by the total number of cycles *K* yields the overall complexity for this recursive loop. Within each cycle, the following operations are executed sequentially:

(i) **Data association (Algorithm 2, Line 6).** In the data association step, the algorithm first constructs an $N_{i}\times N_{t}$ cost matrix with a complexity of $O\left(N_{i}\cdot N_{t}\right)$. After the cost matrix is assembled, the optimal assignment problem is solved using Equation (28). For an $N_{i}\times N_{t}$ assignment, it performs at most $\min\left(N_{i},N_{t}\right)$ steps, and each step scans the entire cost matrix. Thus, the computational cost of the assignment is $O\left(N_{i}\cdot N_{t}\cdot \min\left(N_{i},N_{t}\right)\right)$. Combining these two sequential operations via the addition rule, the overall per-frame complexity of the data association step is $O\left(N_{i}\cdot N_{t}\cdot \min\left(N_{i},N_{t}\right)\right)$.

(ii) **State estimation (Algorithm 2, Line 7).** The complexity of state estimation per time step is $O\left(N_{t}\cdot n^{3}\right)$, whose specific calculation process is similar to pre-maneuver EKF propagation.

(iii) **EM-based spatial bias estimation (Algorithm 2, Lines 8–13).** In the expectation step, the algorithm evaluates the conditional expectation given in Equation (35), which requires a cost of $O\left(l_{EM}\cdot N_{i}\cdot N_{t}\right)$. In the maximization step, the spatial bias update in Equation (38) also carries a cost of $O\left(l_{EM}\cdot N_{i}\cdot N_{t}\right)$. With $N_{EM}$ iterations of the EM algorithm, the total cost of the bias estimation stage is $O\left(N_{EM}\cdot l_{EM}\cdot N_{i}\cdot N_{t}\right)$.

Summing the per-frame costs of data association and EKF updates over the entire main loop, and adding the one-time EM batch cost, the total computational burden of data association and state estimation is expressed as

(40) $$O\left(K\cdot \left[N_{i}\cdot N_{t}\cdot \min\left(N_{i},N_{t}\right)+N_{t}\cdot n^{3}\right]+N_{EM}\cdot l_{EM}\cdot N_{i}\cdot N_{t}\right).$$

Aggregating the costs from all three phases, the overall complexity of the MDA algorithm is given by

(41) $$\begin{array}{cc} O & \left(N_{t}\cdot N_{iter}\cdot L\cdot N_{GN}\cdot \left(n^{2}K+n^{3}\right)+N_{t}\cdot k_{m}\cdot n^{3}+K\cdot \left[N_{i}\cdot N_{t}\cdot \min\left(N_{i},N_{t}\right)+N_{t}\cdot n^{3}\right]\right.\\ \; & \left.+N_{EM}\cdot l_{EM}\cdot N_{i}\cdot N_{t}\right). \end{array}$$

## 5. Simulation and Analysis

### 5.1. Simulation Scenario I

#### 5.1.1. Scenario I Description

**Figure 1:**
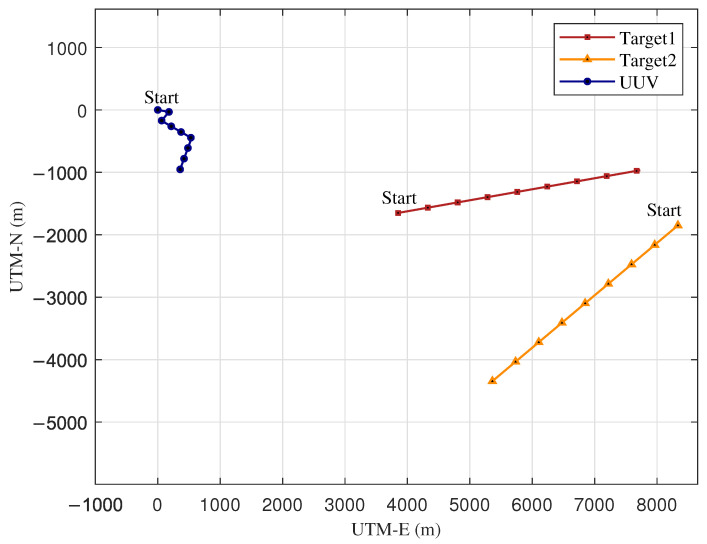
Trajectories of targets and UUV in simulation scenario I.

The validity and effectiveness of the proposed MDA algorithm have been assessed through Monte Carlo simulations. As illustrated in Figure 1, all simulations are conducted within a Cartesian coordinate system, with the initial position of the UUV defined as the origin of the coordinate system. The corresponding simulation parameters along with the initial states of the UUV and targets are detailed in Table 1. During the simulation scenario I, a total of four heading maneuvers are executed by the UUV. The first two maneuvers are performed at intervals of every 60 s, followed by the latter two at intervals of every 180 s.

#### 5.1.2. Algorithm Initialization

The initialization parameters employed in the MDA are presented in Table 2. The time-varying acceleration coefficients govern the cognitive and social trade-off, progressively shifting the search emphasis from individual exploration to swarm exploitation. The inertia weight preserves prior motion trends to maintain global search diversity and prevent abrupt oscillations in the late stage. Additionally, the coupling factor acts as a dimensional scaling vector, which separately moderates the update strides for the target’s velocity and course to compensate for their inherently distinct physical ranges and search space topologies.

At time k=1, the distance between the *j*th target and the UUV follows $r_{j}$∼$N\left({\bar{r}}_{j},\sigma_{r}^{2}\right)$, where ${\bar{r}}_{j}$ denotes the initial estimated distance. The velocity of the *j*th target in the NED coordinate system is distributed as $v_{j}$∼$N\left({\bar{v}}_{j},\sigma_{v}^{2}\right)$, with ${\bar{v}}_{j}$ representing the prior-based velocity estimation. Furthermore, the course of the *j*th target follows $\psi_{j}$∼$N\left({\bar{\psi }}_{j},\sigma_{\psi }^{2}\right)$, where ${\bar{\psi }}_{j}$ is assigned as the initial bearing between the *j*th target and the UUV. Consequently, the mean and covariance of the initial state for the *j*th target can be expressed as

(42) $$\begin{array}{cc} {\hat{X}}_{j}\left(1\right)= & {\left[x\left(1\right)+r_{j}\sin\left(\psi_{j}\right)\;y\left(1\right)+r_{j}\cos\left(\psi_{j}\right)\;v_{j}\sin\left(\psi_{j}\right)\;v_{j}\cos\left(\psi_{j}\right)\right]}^{T}, \end{array}$$

(43) $$\begin{array}{cc} P_{j}\left(1,1\right)=diag & \left[{\sigma_{r}}^{2}\sin^{2}\left(\psi_{j}\right)\;{\sigma_{r}}^{2}\cos^{2}\left(\psi_{j}\right)\;{\sigma_{v}}^{2}\sin^{2}\left(\psi_{j}\right)\;{\sigma_{v}}^{2}\cos^{2}\left(\psi_{j}\right)\right]. \end{array}$$

The joint data association, bias compensation and fusion (JACF) method [23] enables online data association with integrated bias compensation, while the JPDA based on the noise interaction cubature Kalman filter (JPDA-ICKF) [32] addresses association through a joint probabilistic filter. Nevertheless, in BOT scenarios where the measurement model comprises only bearing information, the absence of range data can impair the association accuracy of both methods, particularly when accurate initial target states are unavailable, ultimately leading to performance degradation. To mitigate potential unfairness in benchmark comparisons arising from initial target state uncertainty, the proposed PSO-based initial state estimation algorithm is incorporated into the initialization stage of JACF and JPDA-ICKF, yielding enhanced variants designated as JACF-MLE and JPDA-ICKF-MLE, respectively. To assess the efficacy of the EM component within the MDA, a variant without EM, designated as MDA-NEM, is introduced as a benchmark algorithm in the simulation.

#### 5.1.3. Performance Analysis of Scenario I

To ensure statistical robustness and a fair comparison, all experiments are conducted over 100 Monte Carlo runs. The following parameter configurations are adopted: the maximum iteration number for the precise initial target state estimation algorithm based on PSO and the EM approach within MDA is set to 100; the sample size for EM estimation, denoted as $l_{EM}=K_{i}-k_{i}$, is set to 50. Furthermore, the validation gate threshold (Υ) is set to $2.25e^{-2}$ for the MDA-NEM, MDA, JACF-MLE and JACF (equating to the parameter γ in Equation (17) of [23]). All remaining parameters for the benchmark JACF (including JACF-MLE) and JPDA-ICKF (including JPDA-ICKF-MLE) algorithms adhere to the specifications in [23] and [32], respectively.

**Figure 2:**
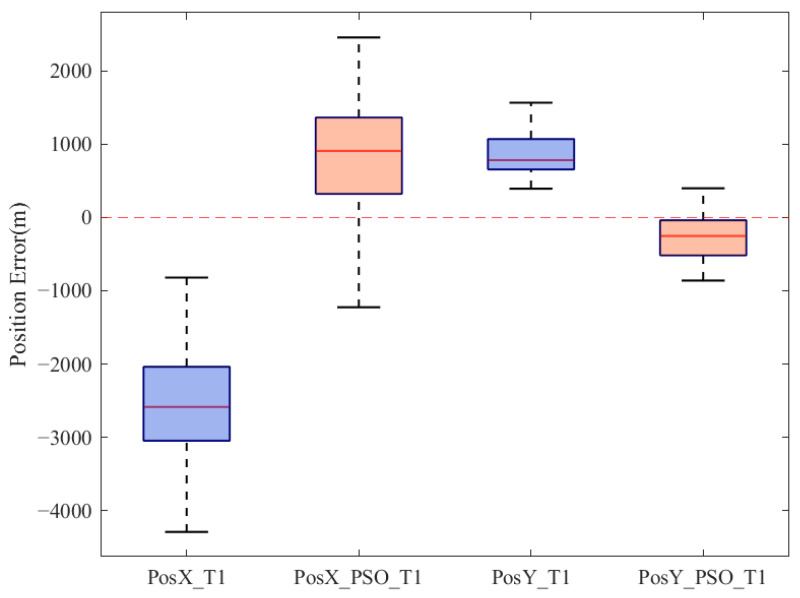
Initial position estimation error distribution for 1st target in simulation scenario I.

**Figure 3:**
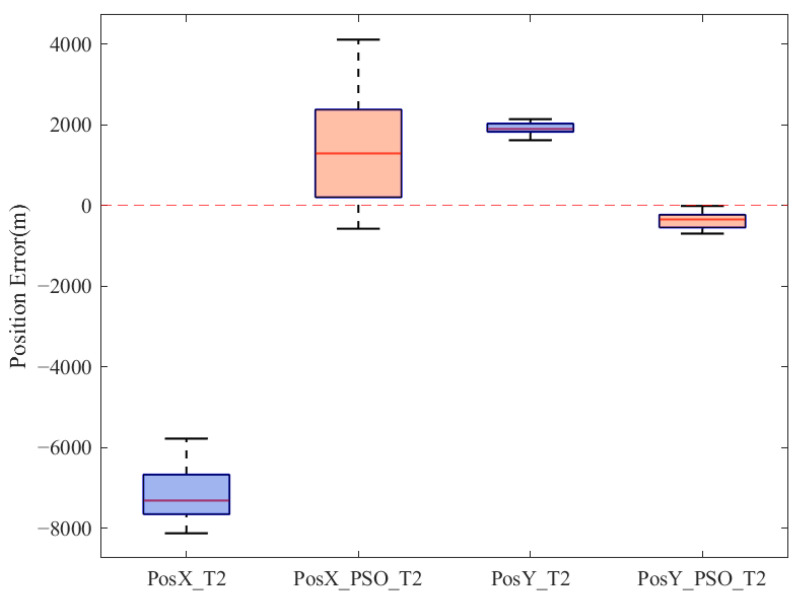
Initial position estimation error distribution for 2nd target in simulation scenario I.

During the first leg of simulation scenario I, the primary objective of the UUV is to minimize the uncertainty of the initial states of targets to the greatest possible extent. Figure 2 and Figure 3 present a comparative analysis of the estimation error distribution for the initial target state estimation algorithms, operating without and with signal delay compensation, respectively. “PSO” refers to a precise initial target state estimation optimization algorithm that explicitly accounts for signal delay compensation. The incorporation of signal delay compensation in the proposed PSO-based algorithm yields a marked improvement over the delay neglecting approach, as evidenced by superior performance across all evaluated metrics (mean, minimum, maximum and quartiles) in the positional error distribution box plots for both targets. This demonstrates the capability of the proposed algorithm in reducing initial target state uncertainty, which, in turn, provides a more reliable foundation for the ensuing data association.

To evaluate the performance of the data association process, the Root Mean Square Error (RMSE) is utilized. This metric quantifies the average magnitude of estimation errors, offering a direct assessment of accuracy and is mathematically defined as

(44) $$\mathrm{RMSE}\left(X_{j}\left(k\right),{\hat{X}}_{j}\left(k\right)\right)=\sqrt{\frac{1}{M}\sum_{m=1}^{M}{\left(X_{j}^{m}\left(k\right)-{\hat{X}}_{j}^{m}\left(k\right)\right)}^{2}},$$

where *M* denotes the total number of Monte Carlo runs, while $X_{j}^{m}\left(k\right)$ and ${\hat{X}}_{j}^{m}\left(k\right)$ are the ground-truth state and the estimated state of the *j*th target at time step *k* in the *m*th run, respectively.

The Cramér–Rao Lower Bound (CRLB) serves as the theoretical benchmark for performance evaluation, representing the fundamental limit on the RMSE achievable by any unbiased approach.

(45) $${\mathrm{CRLB}}_{j}\left(k\right)={\left[\sum_{t=1}^{k}{\left(L_{j}\left(t\right)\right)}^{T}{R_{U}}^{-1}L_{j}\left(t\right)\right]}^{-1},$$

where

(46) $$L_{j}\left(t\right)=\frac{d}{dX_{j}\left(t\right)}\left(\left(z_{U}^{i}\left(k\right)-h\left(X_{U}\left(k\right),X_{j}\left(k\right)\right)\right)\right).$$

**Figure 4:**
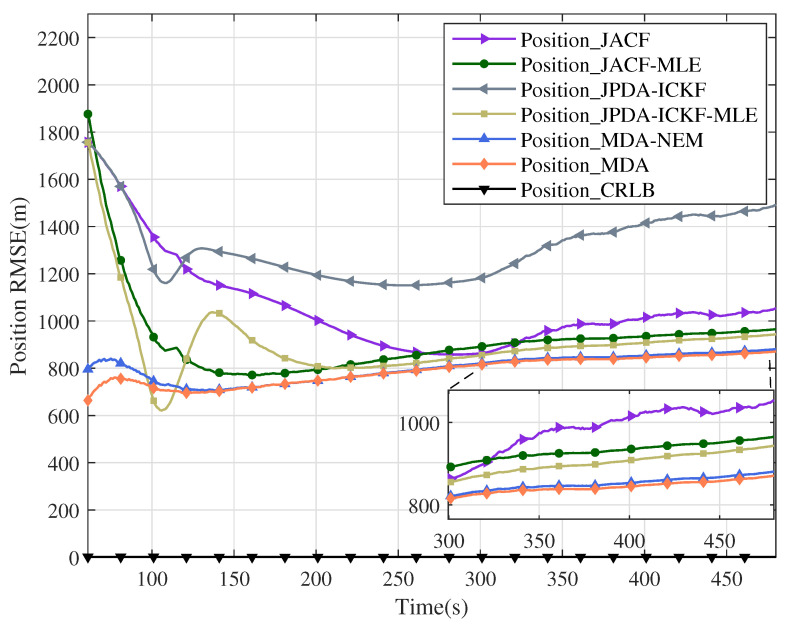
Position RMSE of 1st target versus time in simulation scenario I.

**Figure 5:**
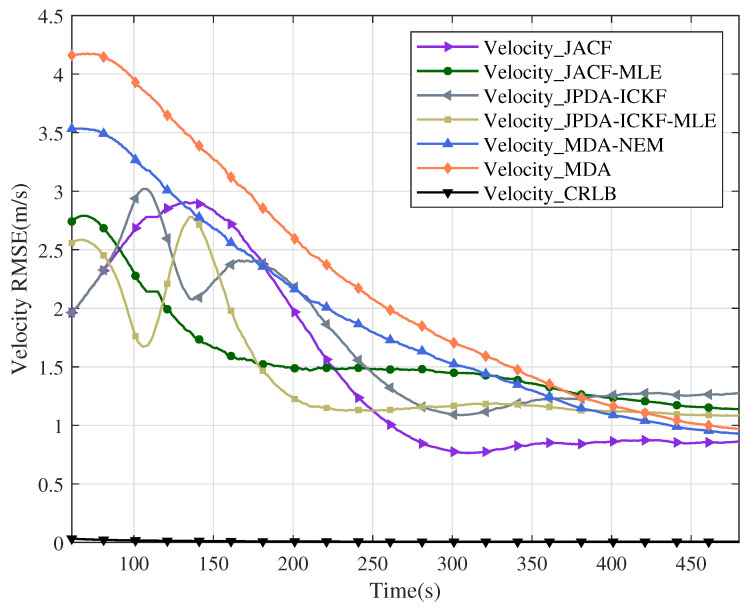
Velocity RMSE of 1st target versus time in simulation scenario I.

As shown in Figure 4 and Figure 5, the improvement in position RMSE curves of JACF-MLE and JPDA-ICKF-MLE over JACF and JPDA-ICKF validates the positive effect of the proposed PSO-based initial state estimation algorithm on state estimation. Under comparable estimation accuracy for the velocity of the first target, the proposed MDA (including MDA-NEM) achieves faster convergence and higher precision in estimating the position of the first target. This result validates the effectiveness of the measurement model incorporating signal delay. In addition, the effectiveness of the proposed EM method for spatial bias estimation is demonstrated by the MDA’s superior position estimation performance relative to the MDA-NEM.

**Figure 6:**
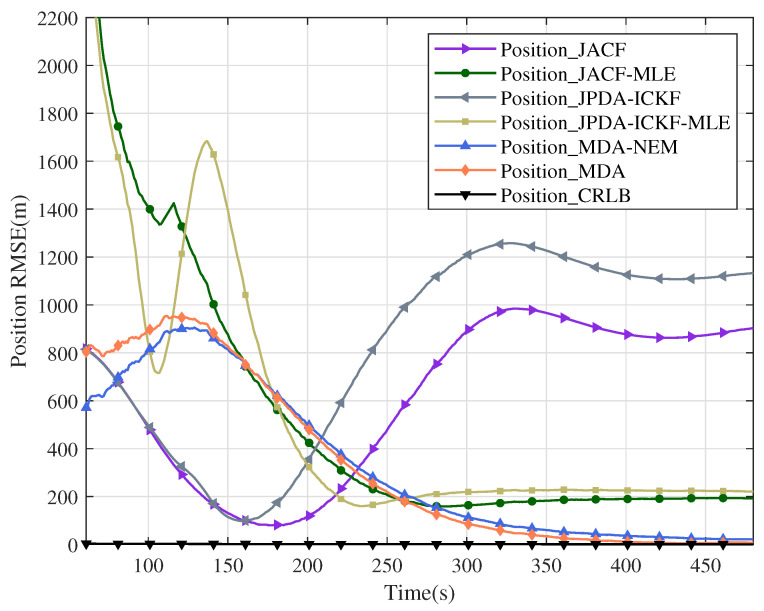
Position RMSE of 2nd target versus time in simulation scenario I.

**Figure 7:**
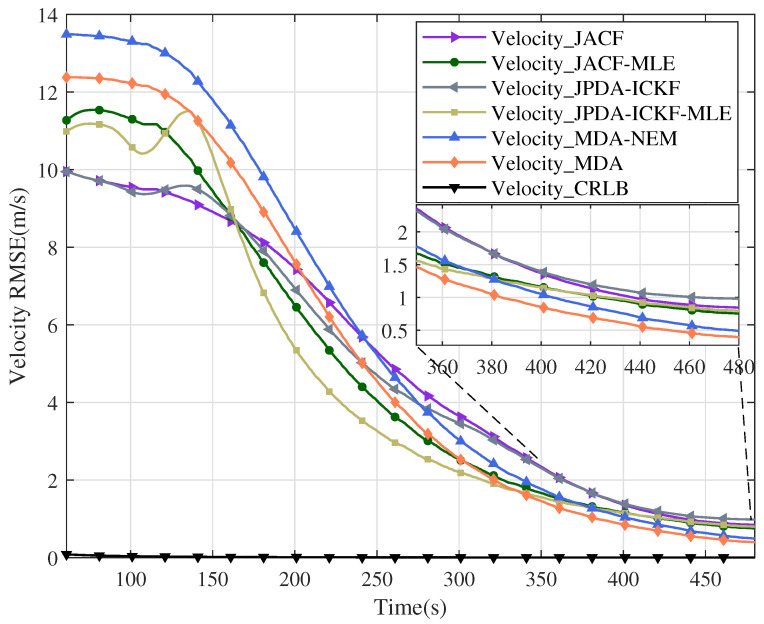
Velocity RMSE of 2nd target versus time in simulation scenario I.

**Figure 8:**
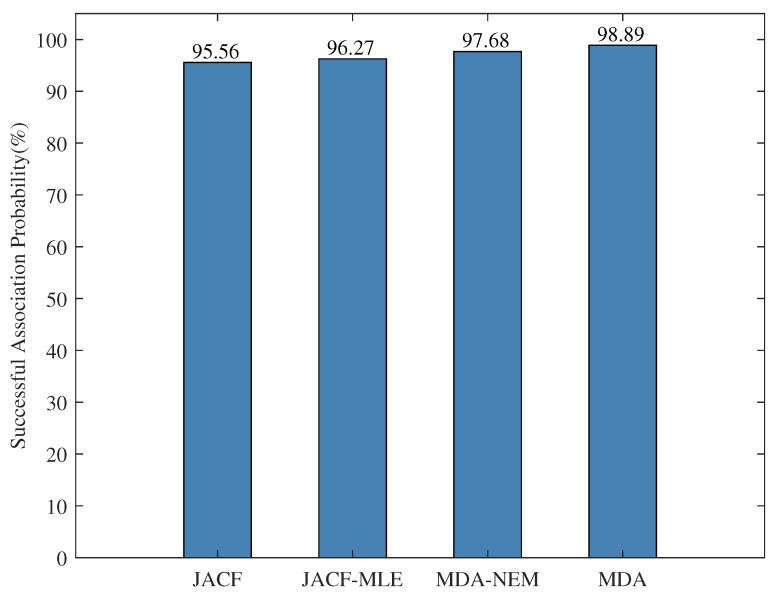
Successful association rate for different methods in simulation scenario I.

**Figure 9:**
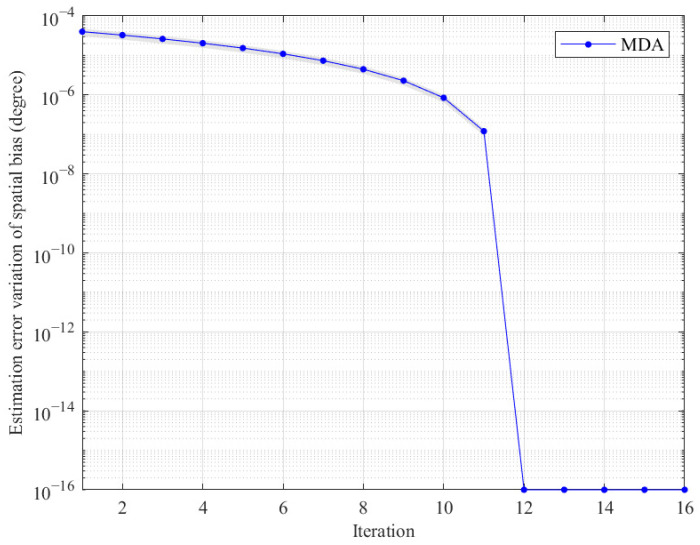
Estimation error variation of spatial bias in simulation scenario I.

Referring back to Figure 3, the proposed PSO-based initial state estimation algorithm provides a better initial covariance matrix, which reflects the uncertainty of the estimated target state. According to Figure 6 and Figure 7, although this reasonable covariance may lead to conservative convergence in early stages, it ultimately enables JACF-MLE and JPDA-ICKF-MLE to achieve smoother and more consistent filtering, yielding higher accuracy after convergence compared to JACF and JPDA-ICKF. MDA-NEM exhibits an advantage over JACF-MLE and JPDA-ICKF-MLE across the RMSE of the position for the second target. The notable accuracy in position convergence, in particular, confirms the impact of the signal delay between the target and the UUV. Correspondingly, the further improvement in estimation accuracy for both the position and velocity achieved by MDA over MDA-NEM validates the efficacy of the proposed EM method in spatial bias estimation. As demonstrated in Figure 8, the high successful association rate achieved by MDA validates the effectiveness of the proposed measurement association cost function. To evaluate the appropriateness of the pre-defined sample count in Section 5.1.3, Table 3 benchmarks the corresponding spatial bias estimation performance. Based on the trade-off between the estimated accuracy and computational cost, 50 samples represent the optimal compromise. Table 4 presents the computational time per run of different algorithms, in which the computational times of both the MDA-NEM and MDA algorithms are shorter than the sampling interval, thereby verifying the real-time capability of the proposed algorithms. As shown in Figure 9, the blue curve represents the median of the MDA algorithm spatial bias estimation error over iteration steps, and the shaded gray region spans the 25th to 75th percentiles. Both converge progressively to lower error levels as iterations proceed, indicating that the convergence process of the EM method is consistent.

Table 5 and Table 6 present the tracking performance under varying levels of measurement noise. As can be seen from Table 5, compared with the comparative algorithms, MDA achieves a comparable convergence time and velocity RMSE for the first target while reducing the position RMSE by at least 18 m. For the second target, MDA reduces the convergence time by at least 23 s, the position RMSE by at least 97 m and the velocity RMSE by at least 0.30 m/s. As shown in Table 6, compared with the comparative algorithms, MDA achieves a comparable convergence time and velocity RMSE for the first target while reducing the position RMSE by at least 27 m. For the second target, MDA reduces the convergence time by at least 39 s, while the position RMSE and velocity RMSE show no significant difference.

A comparative analysis reveals that, under conditions of increased measurement noise, the convergence time and overall performance of the algorithms generally degrade. However, the PSO-based initial state estimation algorithm proposed in this paper mitigates the uncertainty in target state initialization through heuristic search during state estimation. This process establishes a more robust foundation for subsequent target association and estimation. Consequently, this explains the improvement in state estimation performance for the first target despite the heightened measurement noise.

### 5.2. Simulation Scenario II

#### 5.2.1. Scenario II Description

**Figure 10:**
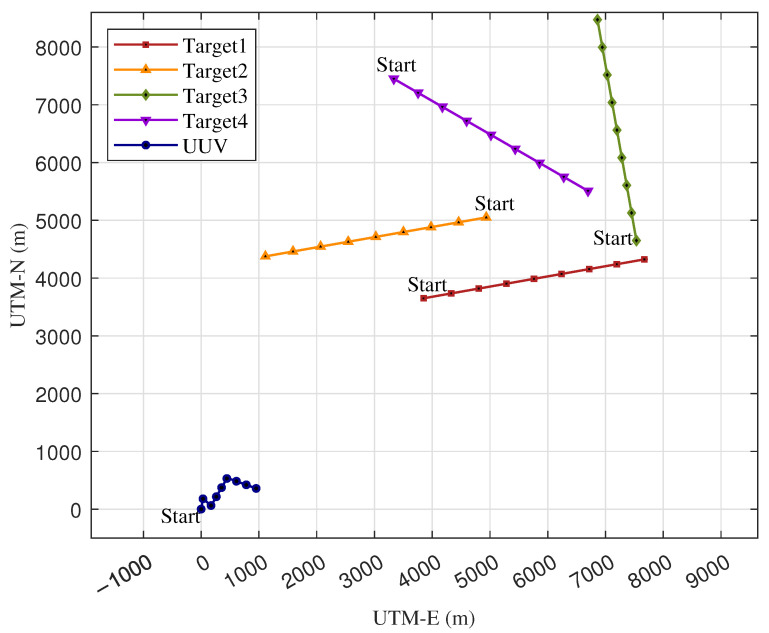
Trajectories of targets and UUV in simulation scenario II.

To further validate the effectiveness and performance of the proposed MDA algorithm under non-Gaussian noise, Figure 10 illustrates the trajectories of the UUV and targets in Monte Carlo simulation scenario II. The simulation parameters related to the UUV and target states are detailed in Table 7.

The number of Monte Carlo simulation runs, as well as the initialization and parameter settings of all methods, remain consistent with those in scenario I. To validate the robustness of all those methods, the mean of the initial distance between the UUV and target is extended to ${\bar{r}}_{j}=$ 10,000 m. Moreover, two Gaussian noise mixing models are employed to simulate non-Gaussian measurement noise, which is modeled as

(47) $$\begin{array}{cc} V\left(k\right)∼\left\{\begin{array}{c} N\left(0,R_{U}\right)\\ N\left(0,100R_{U}\right) \end{array}\right. & \begin{array}{c} w.p.\;0.9\\ w.p.\;0.1 \end{array} \end{array}.$$

#### 5.2.2. Performance Analysis of Scenario II

**Figure 11:**
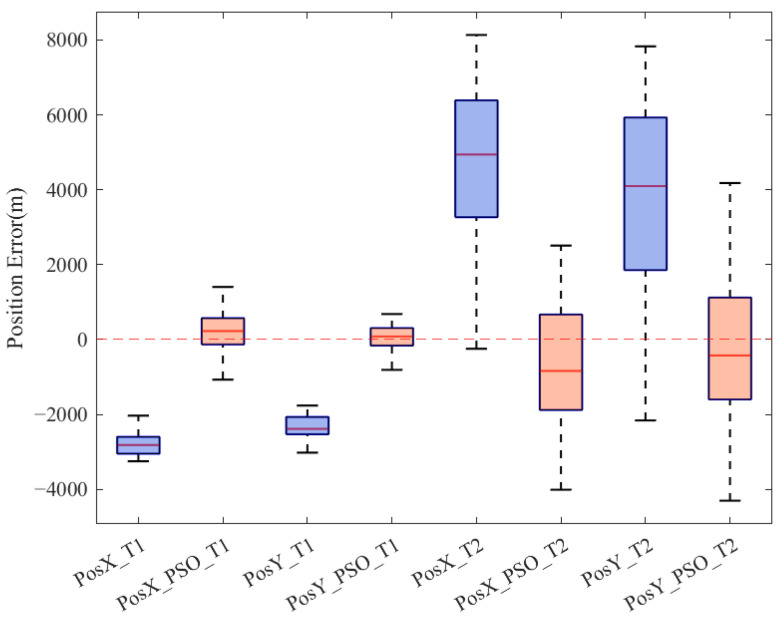
Initial position estimation error distribution for 1st target and 2nd target in simulation scenario II.

**Figure 12:**
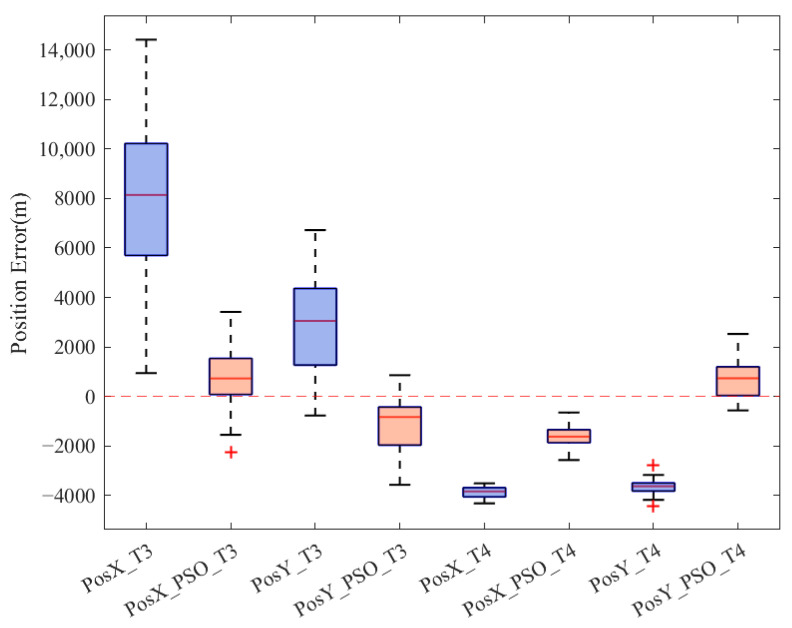
Initial position estimation error distribution for 3rd target and 4th target in simulation scenario II.

According to Figure 11 and Figure 12, under increased initial target state uncertainty and non-Gaussian measurement noise, the proposed algorithm nevertheless achieves superior performance, thereby laying a solid foundation for the subsequent data association process.

**Figure 13:**
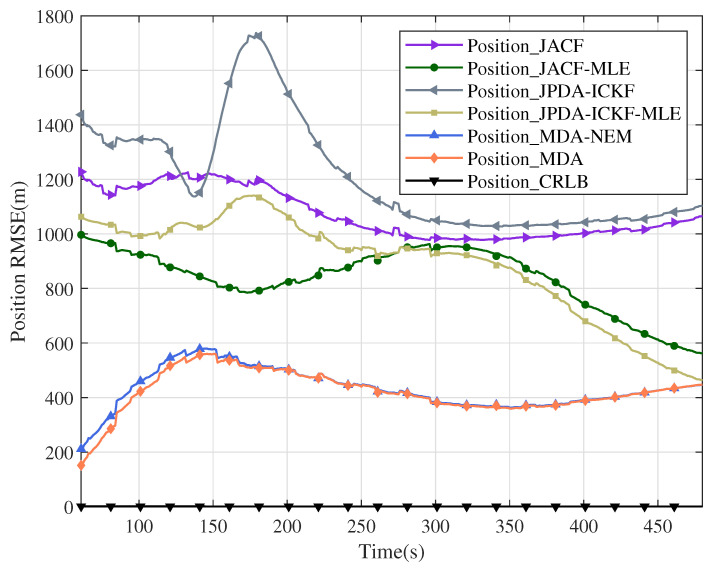
Position RMSE of 1st target versus time in simulation scenario II.

**Figure 14:**
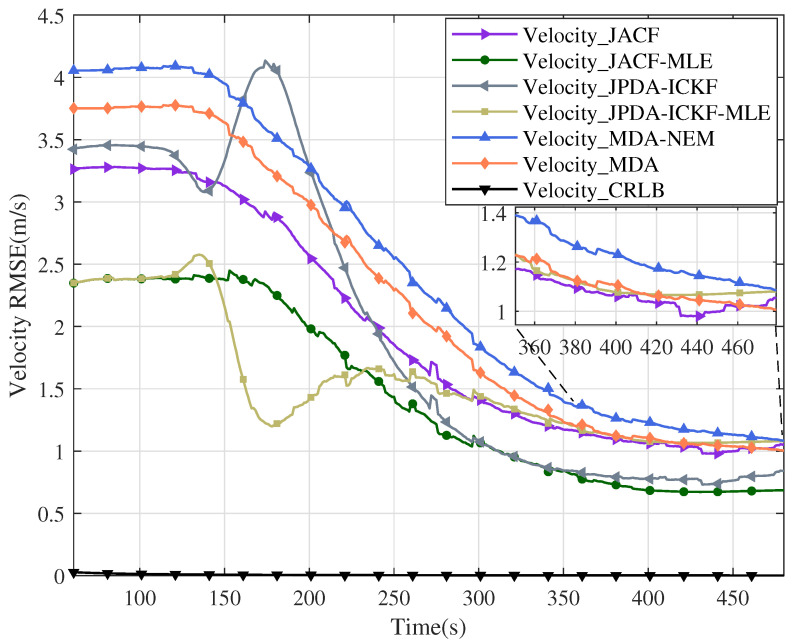
Velocity RMSE of 1st target versus time in simulation scenario II.

**Figure 15:**
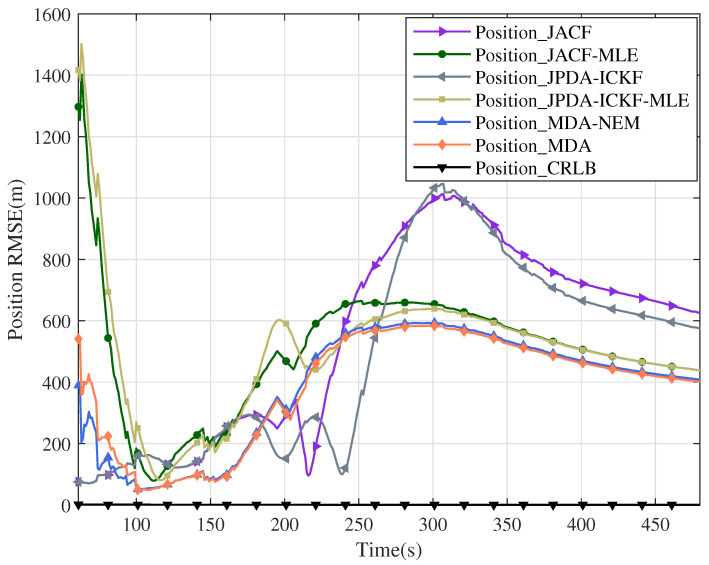
Position RMSE of 2nd target versus time in simulation scenario II.

**Figure 16:**
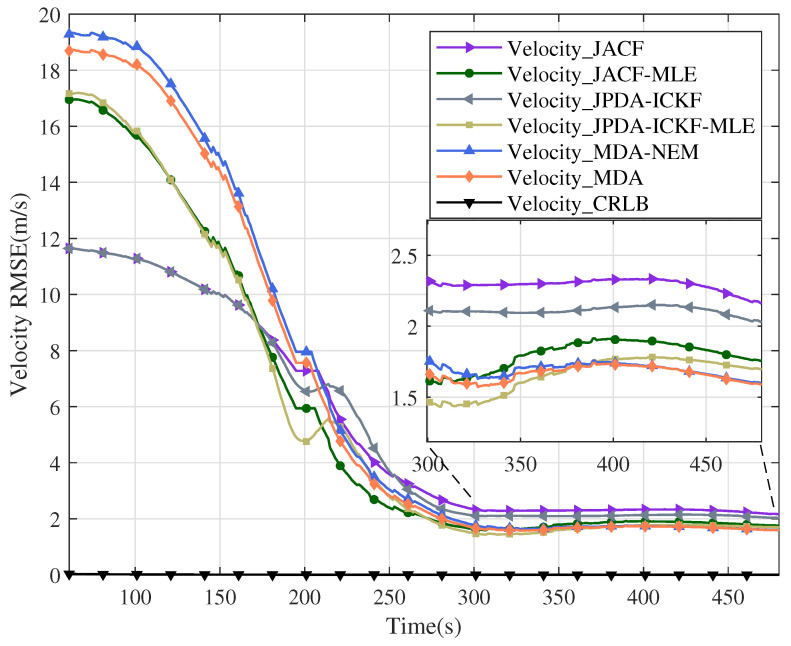
Velocity RMSE of 2nd target versus time in simulation scenario II.

**Figure 17:**
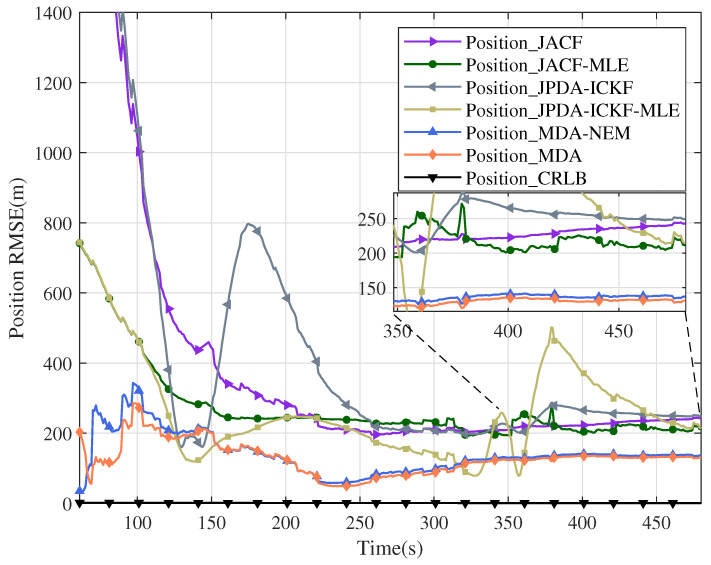
Position RMSE of 3rd target versus time in simulation scenario II.

**Figure 18:**
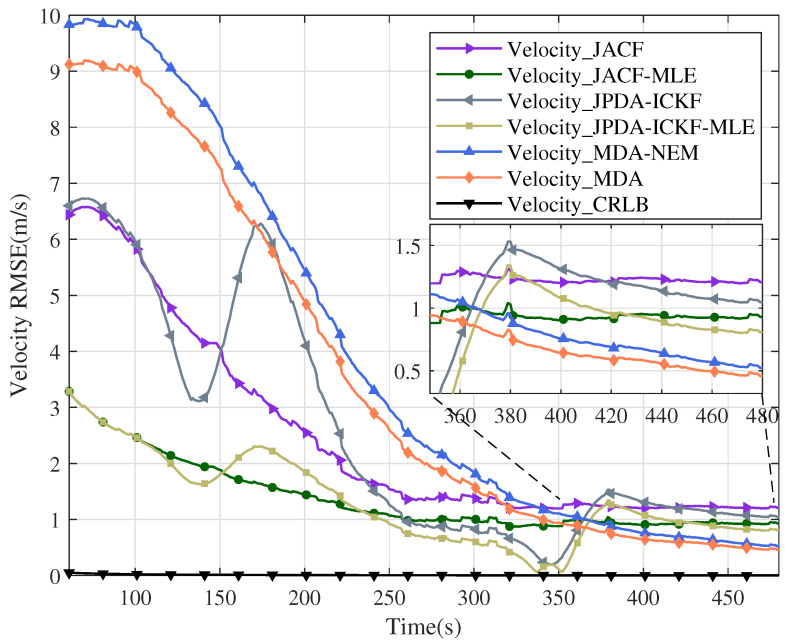
Velocity RMSE of 3rd target versus time in simulation scenario II.

**Figure 19:**
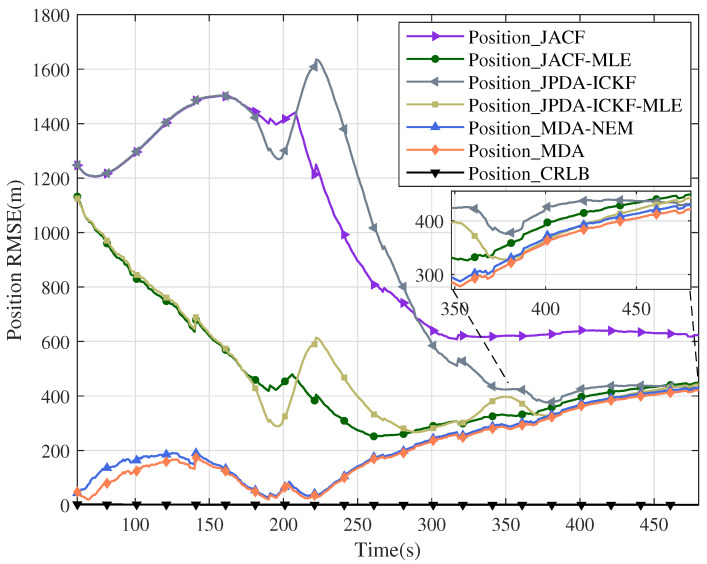
Position RMSE of 4th target versus time in simulation scenario II.

**Figure 20:**
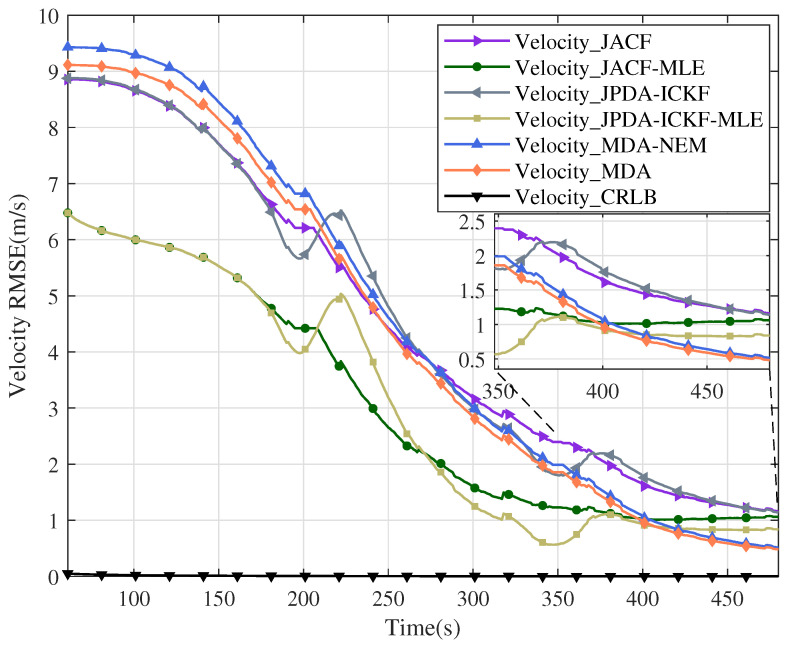
Velocity RMSE of 4th target versus time in simulation scenario II.

**Figure 21:**
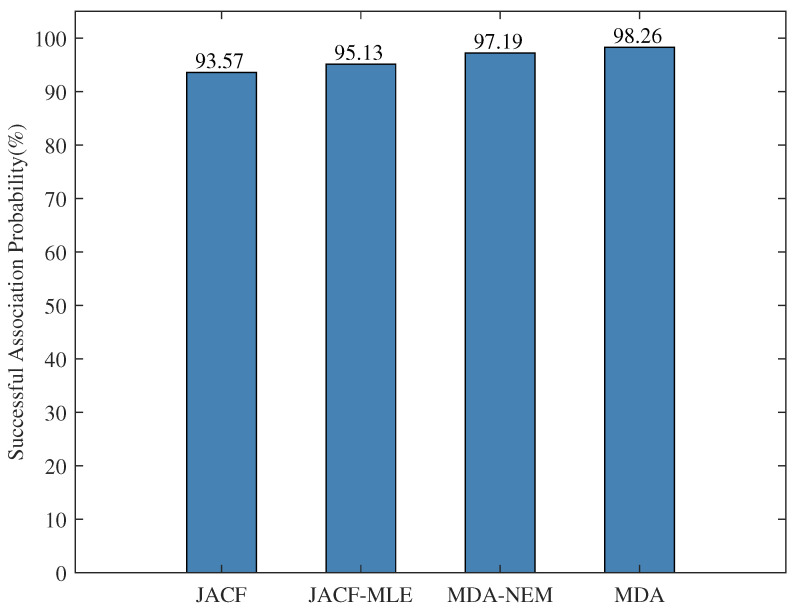
Successful association rate for different methods in simulation scenario II.

**Figure 22:**
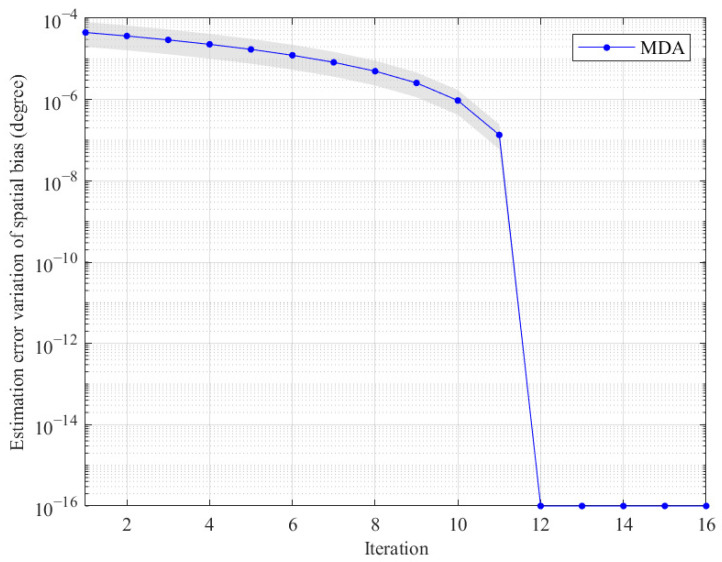
Estimation error variation of spatial bias in simulation scenario II.

Figure 13, Figure 14, Figure 15, Figure 16, Figure 17, Figure 18, Figure 19 and Figure 20 present the estimated target state RMSE curves obtained by different methods. JPDA-ICKF and JPDA-ICKF-MLE exhibit several pronounced fluctuations, particularly in Figure 17 and Figure 18. This is attributable to the fact that JPDA-ICKF and JPDA-ICKF-MLE perform weighted fusion of all candidate measurements through association probabilities. When erroneous measurements are assigned elevated weights, the resulting prediction error propagates to the subsequent time step, further diminishing the association weights of true measurements and thereby forming a positive feedback drift. Consequently, the filter continues to converge toward erroneous sources and can only recover gradually after the UUV maneuvers or covariance inflation occurs. MDA-NEM exhibits overall superior performance compared to JACF and JPDA-ICKF, particularly during the beginning stage of the position RMSE curves, reflecting the positive impact brought by the improvement in initial target state estimation. Figure 21 demonstrates the superiority of MDA in the successful association rate. Figure 22 illustrates the convergence performance of the EM method.

As can be seen from Table 8, compared with the comparative algorithms, MDA reduces the position RMSE of the first target by at least 105 m while maintaining a comparable convergence time and velocity RMSE. For the second target, MDA reduces the position RMSE by at least 37 m, with the convergence time and velocity RMSE remaining at a similar level. As shown in Table 9, compared with the comparative algorithms, MDA reduces the position RMSE of the third target by at least 139 m and the velocity RMSE by at least 0.35 m/s, while the convergence time remains comparable. For the fourth target, MDA reduces the position RMSE by at least 17 m and the velocity RMSE by at least 0.36 m/s, with convergence time again showing no significant difference. Furthermore, the accuracy improvement of MDA over MDA-NEM demonstrates the effectiveness of the EM method.

## 6. Conclusions

Focusing on the challenges of underwater bearing-only tracking, this paper introduces a series of interconnected solutions. The core innovation begins with a measurement model that explicitly considers spatial bias and signal delay. To tackle the uncertainty of state initialization, a signal delay compensated MLE algorithm is proposed, which is further enhanced by a PSO-based variant. For the critical data association step, it is recast as an assignment problem solved via a specifically designed cost function. Additionally, potential sensor bias is mitigated through an EM-based spatial bias estimation method. Comprehensive Monte Carlo simulation scenarios demonstrate that the MDA algorithm outperforms the compared algorithms. Extending this work, future efforts will integrate target maneuverability models with a strong emphasis on data-driven algorithms to address the ensuing association complexity.

## Figures and Tables

**Table 1:** Specifications of simulation scenario I.

Name	Symbol	Parameter
1st Target Initial Position (m)	$\left(x_{1}\left(1\right),y_{1}\left(1\right)\right)$	(3850,−1650)
1st Target Velocity (knots)	$\left({\dot{x}}_{1}\left(1\right),{\dot{y}}_{1}\left(1\right)\right)$	(15.74,2.78)
2nd Target Initial Position (m)	$\left(x_{2}\left(1\right),y_{2}\left(1\right)\right)$	(8335,−1850)
2nd Target Velocity (knots)	$\left({\dot{x}}_{2}\left(1\right),{\dot{y}}_{2}\left(1\right)\right)$	(−12.25,−10.28)
UUV Initial Position (m)	(x(1),y(1))	(0,0)
UUV Velocity of First Leg (knots)	$\left(\dot{x}\left(K^{1}\right),\dot{y}\left(K^{1}\right)\right)$	(5.90,−1.04)
Duration of UUV First Leg (s)	$K^{1}$	[1,60]
UUV Velocity of Second Leg (knots)	$\left(\dot{x}\left(K^{2}\right),\dot{y}\left(K^{2}\right)\right)$	(−3.85,−4.59)
Duration of UUV Second Leg (s)	$K^{2}$	[61,120]
UUV Velocity of Third Leg (knots)	$\left(\dot{x}\left(K^{3}\right),\dot{y}\left(K^{3}\right)\right)$	(5.29,−3.00)
Duration of UUV Third Leg (s)	$K^{3}$	[121,300]
UUV Velocity of Fourth Leg (knots)	$\left(\dot{x}\left(K^{4}\right),\dot{y}\left(K^{4}\right)\right)$	(−2.05,−5.63)
Duration of UUV Fourth Leg (s)	$K^{4}$	[301,480]
Distribution of Underwater Acoustic Signal Speed (m/s)	$c\left(k\right)=N\left(c,\sigma_{c}^{2}\right)$	$N\left(1450,10\right)$
Sampling Interval (s)	$\Delta T_{U}$	1
Total Simulation Time (s)	$K_{i}$	480
Distribution of Spatial Bias (degree)	$\Delta \theta_{U}^{i}\left(k\right)$	$N\left(0.2,0.01\right)$

**Table 2:** Initialization parameters of MDA.

Name	Symbol	Parameter
Power Spectral Density of Process Error (degree)	*q*	0.01
Variance of Measurement Noise (degree^2^)	$R_{U}$	0.04
Mean of Initial Distance (m)	${\bar{r}}_{j}$	6500
Standard Deviation of Initial Distance (m)	$\sigma_{r}$	2000
Target Maximum Velocity (knots)	$v_{j\max}$	20
Mean of Initial Target Velocity (knots)	${\bar{v}}_{j}$	18
Standard Deviation of Initial Target Velocity (knots)	$\sigma_{v}$	5
Standard Deviation of Initial Target Course (degree)	$\sigma_{\psi }$	10
Initial Spatial Bias Estimation (degree)	$\Delta {\hat{\theta }}_{U}^{i}\left(k\right)$	1
Maximum Number of Iterations	$N_{iter}$	15
Population Size of Particles	*L*	60
Inertia Weights	$\alpha^{1}={\left[\begin{array}{cc} \alpha_{V}^{1} & \alpha_{\psi }^{1} \end{array}\right]}^{T}$	${\left[\begin{array}{cc} 0.30 & 0.70 \end{array}\right]}^{T}$
Acceleration Coefficients	${\left[\begin{array}{cc} \beta^{min} & \beta^{max} \end{array}\right]}^{T}$	${\left[\begin{array}{cc} 0.50 & 2.50 \end{array}\right]}^{T}$
Acceleration Coefficients	${\left[\begin{array}{cc} \gamma^{min} & \gamma^{max} \end{array}\right]}^{T}$	${\left[\begin{array}{cc} 0.50 & 2.00 \end{array}\right]}^{T}$
Coupling Factors	${\left[\begin{array}{cc} \mu_{V} & \mu_{\psi } \end{array}\right]}^{T}$	${\left[\begin{array}{cc} 0.01 & 0.18 \end{array}\right]}^{T}$

**Table 3:** EM estimation performance with different number of samples in simulation scenario I.

Number of Samples	Error of Estimated Spatial Bias (Degree)	Computation Time (ms)
30	1.293	35
50	0.064	42
80	0.063	49

**Table 4:** Computation time of different methods in simulation scenario I.

Method	Computation Time (ms)
JACF	7
JPDA-ICKF	8
MDA-NEM	4
MDA	4 (46 *)

* When EM estimation is introduced.

**Table 5:** Tracking performance with measurement noise ($R_{U}$ = 0.04) in simulation scenario I.

Method	Convergent Time of 1st Target (s)	Position RMSE of 1st Target (m)	Velocity RMSE of 1st Target (m/s)	Convergent Time of 2nd Target (s)	Position RMSE of 2nd Target (m)	Velocity RMSE of 2nd Target (m/s)
JACF	292	859	0.85	370	878	0.93
JACF-MLE	279	855	1.46	362	165	0.84
JPDA-ICKF	299	1179	1.26	369	1127	1.02
JPDA-ICKF-MLE	276	817	1.23	351	219	0.81
MDA-NEM	245	780	0.95	344	84	0.64
MDA	277	799	0.99	328	68	0.51

**Table 6:** Tracking performance with measurement noise ($R_{U}$ = 0.15) in simulation scenario I.

Method	Convergent Time of 1st Target (s)	Position RMSE of 1st Target (m)	Velocity RMSE of 1st Target (m/s)	Convergent Time of 2nd Target (s)	Position RMSE of 2nd Target (m)	Velocity RMSE of 2nd Target (m/s)
JACF	218	1088	0.98	440	817	1.41
JACF-MLE	215	429	0.47	437	862	1.39
JPDA-ICKF	272	1135	1.35	431	1192	1.48
JPDA-ICKF-MLE	267	527	0.79	426	1105	1.42
MDA-NEM	210	417	0.42	399	811	1.39
MDA	193	402	0.39	387	834	1.34

**Table 7:** Specifications of simulation scenario II.

Name	Symbol	Parameter
1st Target Initial Position (m)	$\left(x_{1}\left(1\right),y_{1}\left(1\right)\right)$	(3850,3650)
1st Target Velocity (knots)	$\left({\dot{x}}_{1}\left(1\right),{\dot{y}}_{1}\left(1\right)\right)$	(15.74,2.78)
2nd Target Initial Position (m)	$\left(x_{2}\left(1\right),y_{2}\left(1\right)\right)$	(4935,5050)
2nd Target Velocity (knots)	$\left({\dot{x}}_{2}\left(1\right),{\dot{y}}_{2}\left(1\right)\right)$	(−15.74,−2.78)
3rd Target Initial Position (m)	$\left(x_{3}\left(1\right),y_{3}\left(1\right)\right)$	(7535,4650)
3rd Target Velocity (knots)	$\left({\dot{x}}_{3}\left(1\right),{\dot{y}}_{3}\left(1\right)\right)$	(−2.78,15.74)
4th Target Initial Position (m)	$\left(x_{4}\left(1\right),y_{4}\left(1\right)\right)$	(3335,7450)
4th Target Velocity (knots)	$\left({\dot{x}}_{4}\left(1\right),{\dot{y}}_{4}\left(1\right)\right)$	(13.85,−7.99)
UUV Initial Position (m)	(x(1),y(1))	(0,0)
UUV Velocity of First Leg (knots)	$\left(\dot{x}\left(K^{1}\right),\dot{y}\left(K^{1}\right)\right)$	(1.04,5.90)
Duration of UUV First Leg (s)	$K^{1}$	[1,60]
UUV Velocity of Second Leg (knots)	$\left(\dot{x}\left(K^{2}\right),\dot{y}\left(K^{2}\right)\right)$	(4.59,−3.85)
Duration of UUV Second Leg (s)	$K^{2}$	[61,120]
UUV Velocity of Third Leg (knots)	$\left(\dot{x}\left(K^{3}\right),\dot{y}\left(K^{3}\right)\right)$	(2.99,5.19)
Duration of UUV Third Leg (s)	$K^{3}$	[121,300]
UUV Velocity of Fourth Leg (knots)	$\left(\dot{x}\left(K^{4}\right),\dot{y}\left(K^{4}\right)\right)$	(5.63,−2.05)
Duration of UUV Fourth Leg (s)	$K^{4}$	[301,480]
Distribution of Underwater Acoustic Signal Speed (m/s)	$c\left(k\right)=N\left(c,\sigma_{c}^{2}\right)$	$N\left(1450,10\right)$
Sampling Interval (s)	$\Delta T_{U}$	1
Total Simulation Time (s)	$K_{i}$	480
Distribution of Spatial Bias (degree)	$\Delta \theta_{U}^{i}\left(k\right)$	$N\left(0.2,0.01\right)$

**Table 8:** Tracking performance of 1st target and 2nd target in simulation scenario II.

Method	Convergent Time of 1st Target (s)	Position RMSE of 1st Target (m)	Velocity RMSE of 1st Target (m/s)	Convergent Time of 2nd Target (s)	Position RMSE of 2nd Target (m)	Velocity RMSE of 2nd Target (m/s)
JACF	277	983	1.05	282	624	2.16
JACF-MLE	279	562	0.69	281	439	1.75
JPDA-ICKF	276	1046	0.84	308	574	2.03
JPDA-ICKF-MLE	281	466	1.09	275	438	1.71
MDA-NEM	282	363	1.08	302	408	1.60
MDA	261	361	1.01	293	401	1.58

**Table 9:** Tracking performance of 3rd target and 4th target in simulation scenario II.

Method	Convergent Time of 3rd Target (s)	Position RMSE of 3rd Target (m)	Velocity RMSE of 3rd Target (m/s)	Convergent Time of 4th Target (s)	Position RMSE of 4th Target (m)	Velocity RMSE of 4th Target (m/s)
JACF	223	243	1.21	369	622	1.16
JACF-MLE	206	211	0.93	279	271	1.06
JPDA-ICKF	243	249	1.05	375	387	1.14
JPDA-ICKF-MLE	227	214	0.81	275	299	0.84
MDA-NEM	292	89	0.52	316	265	0.52
MDA	278	72	0.46	313	254	0.48

## Data Availability

The original contributions presented in this study are included in this article. Further inquiries can be directed to the corresponding author.
